# A comparative analysis of small extracellular vesicle (sEV) micro-RNA (miRNA) isolation and sequencing procedures in blood plasma samples

**DOI:** 10.20517/evcna.2023.55

**Published:** 2024-02-29

**Authors:** Pevindu Abeysinghe, Natalie Turner, Murray D. Mitchell

**Affiliations:** ^1^Centre for Children’s Health Research, Centre for Immunology and Infection Control, School of Biomedical Sciences, Faculty of Health, Queensland University of Technology, Brisbane, QLD 4101, Australia.; ^2^Centre for Genomics and Personalised Health, School of Biomedical Sciences, Faculty of Health, Queensland University of Technology, Kelvin Grove, QLD 4059, Australia.

**Keywords:** Small extracellular vesicles, exosomes, miRNA, size exclusion chromatography, ultracentrifugation, ultrafiltration, next-generation sequencing, immune function

## Abstract

**Aims:**

Analysis of miRNA (18-23nt) encapsulated in small extracellular vesicles (sEVs) (diameter ~30-200 nm) is critical in understanding the diagnostic and therapeutic value of sEV miRNA. However, various sEV enrichment techniques yield different quantities and qualities of sEV miRNA. Here, we compare the efficacy of three sEV isolation techniques in four combinations for miRNA next-generation sequencing.

**Methods:**

Blood plasma from four Holstein-Friesian dairy cows (*Bos taurus*) (*n* = 4) with similar genetic traits and physical characteristics were pooled to isolate sEV. Ultracentrifugation (UC) (100,000 × *g*, 2 h at 4 °C), size-exclusion chromatography (SEC) and ultrafiltration (UF) were used to design four groups of sEV isolations (UC+SEC, SEC+UC, SEC+UF and UC+SEC+UF). sEV miRNAs were isolated using a combination of TRIzol, Chloroform and miRNeasy mini kit (*n* = 4/each), later sequenced utilizing Novaseq S1 platform (single-end 100 bp sequencing).

**Results:**

All four sEV methods yielded > 1,700 miRNAs and sEV miRNAs demonstrated a clear separation from control blood plasma circulating miRNA (PCA analysis). MiR-381-3p, miR-23-3p, and miR-18b-3p are among the 25 miRNAs unique to sEV, indicating potential sEV-specific miRNA markers. Further, those 25 miRNAs mostly regulate immune-related functions, indicating the value of sEV miRNA cargo in immunology.

**Conclusion:**

The four sEV miRNA isolation methods employed in this study are valid techniques. The choice of method depends on the research question and study design. If purity is of concern, the UC+SEC method resulted in the best particles/µg protein ratio, which is often used as an indication of sample purity. These results could eventually establish sEV miRNAs as effective diagnostic and therapeutic tools of immunology.

## INTRODUCTION

The study of small extracellular vesicles (sEVs) (~30-200 nm) is a topic of interest in immunology, with sEV-encapsulated miRNA being of particular interest. However, there is no consensus on a particular sEV miRNA isolation methodology to facilitate successful miRNA next-generation sequencing. Stand-alone passive EV isolation methods^[1-3]^ or kit-based methods^[4]^ have been utilized to isolate and sequence miRNA from arrays of EV populations (~30-1,000 nm). However, the low abundance of miRNA, specifically in sEVs, poses challenges in the isolation of miRNA from sEVs for next-generation sequencing^[5]^.

There are different strategies for EV isolation such as utilization of active forces: magnetic bead-based^[6]^, acoustophoresis^[7]^, and dielectrophoretic^[8]^-assisted adsorption techniques, which require less time. However, in relation to higher EV purity and subpopulation content, passive EV isolation techniques are commonly selected, even if passive EV isolation techniques are often time-consuming and laborious^[9]^. Ultracentrifugation (UC), which is based on density and hydrodynamics of biofluids, is often referred to as the gold standard of EV isolation^[10]^. However, numerous studies report data contradict this, stating that stand-alone UC techniques yield poor EV particle recovery rates^[11]^ and EV cellular functionality is affected due to particle rupture^[12]^. Size-exclusion chromatography (SEC), which is based on selective particle retention in a porous gel resin and subsequent elution into fractions of equal and pre-defined volumes, enables comparatively rapid EV recovery even with smaller sample volumes. The integrity of EV characteristics was preserved in SEC^[13]^, but EV yield was compromised with more serum protein contaminants compared to UC^[14]^. Ultrafiltration (UF) is a sequential filtration technique to isolate EV which is comparatively cheaper and efficient in terms of time^[15]^ and has been reported to improve EV yield within an appropriate size range compared to UC^[16]^. However, rather than utilizing these three sEV isolation techniques separately, combinatory techniques have been reported to be more successful in sEV enrichment: UF+SEC to avoid serum protein contamination^[17-19]^, UC+SEC to enrich exosomes from blood plasma^[20]^. We have previously reported on the use of the above techniques for downstream proteomic analysis, with UC+SEC resulting in the best purity and highest number of protein identifications using mass spectrometry-based proteomics^[21]^. The broad acceptance and adaptability of these sEV isolation methodologies (UC, SEC, and UF) enable their application and customization to accommodate a variety of sample volumes and types. Hence, we opted for these sEV isolation methods in our study.

Next-generation sequencing (NGS) of miRNA provides a state-of-art characterization technique for miRNA^[4]^, which allows more reliable in-depth profiling in contrast to target-based traditional RT-qPCR^[22]^ or NanoString multiplex miRNA expression assays^[23]^. However, the low abundance of small RNA in sEVs is often a challenge in terms of isolating miRNA of higher quality as required for next-generation sequencing. A recent study has successfully sequenced serum-derived EV miRNAs using NGS platforms utilizing commercially available isolating kits and stand-alone UC for EV isolation, which suggests the efficacy of those EV miRNA isolating pipelines for biomarker discovery^[4]^. More recently, many researchers have attempted to utilize NGS platforms and were successful in characterizing plasma EV-derived miRNAs isolated using UC^[1,24]^ or SEC^[3,25]^ approaches. The objective of the current study was to assess the efficacy of four combinations of sEV miRNA isolating strategies on miRNA next-generation sequencing. Three sEV isolation techniques, UC, SEC, and UF, were utilized to combine into four different combinations (UC+SEC, SEC+UC, SEC+UF, and UC+SEC+UF), and later, an optimized miRNA isolation methodology was used to isolate miRNA from sEV samples. Data generated from NGS platforms were analyzed using an optimized bioinformatics pipeline as well.

## MATERIALS AND METHODS

### Animals, management, and blood collection

The use of animals, management and blood collection were approved by the Raukura Animal Ethics Committee (AEC#14200). Utilization of the Holstein-Friesian primiparous dairy cow model was part of a larger experiment performed by DairyNZ (Tokanui AgResearch), in which animals were managed in a pasture-based, spring-calving dairy system^[26]^. The use of samples was approved by the Queensland University of Technology Animal Ethics Committee (QVR #83807 and #83810). A detailed description of animal inclusion criteria is available in our parallel study by Turner *et al.*^[21]^. For this study, blood plasma samples of dairy cows (*n* = 4) were selected and pooled together from the larger group (*n* = 80) based on similar genetic merit, fertility breeding values (FBV), and physical attributes. The study was conducted in compliance with Australian code for the care and use of animals for scientific purposes^[27]^ and ARRIVE (animal research: reporting of *in vivo* experiments) guidelines, and all methods were performed in accordance with the relevant guidelines and regulations^[28]^.

Blood was collected from animals by coccygeal venepuncture into evacuated EDTA blood tubes and blood was placed on ice and briefly centrifuged for 1,500 × *g* for 12 min at 4 °C immediately upon collection as previously described by Crookenden *et al.*^[29]^. Then, the plasma was aliquoted, frozen, and stored at -80 °C until further experimental procedures conducted for sEV isolation.

### SEV isolation and enrichment

The sEV isolation methodological approaches were similar to our previously published work, which evaluated the proteomic content of sEVs isolated from similar enrichment strategies^[21]^.

Briefly, they are the following three sEV isolation techniques: (1) ultracentrifugation (UC) (100,000 × *g* for 2 h at 4 °C); (2) size-exclusion chromatography (SEC) [70 nm qEV original (Izone Science, Christchurch, New Zealand)] and (3) ultrafiltration (UF) (Amicon Ultra-2 Centrifugal Filter Units (UFC200324, Merck Millipore, Melbourne, Australia)). SEC fractions 7-10 were pooled based on low abundance of cellular plasma contaminant bovine serum albumin (BSA) protein in contrast to fractions 11-16 which showed relatively stronger signal of BSA as mentioned in the previous publication^[21]^.

**Figure 1:**
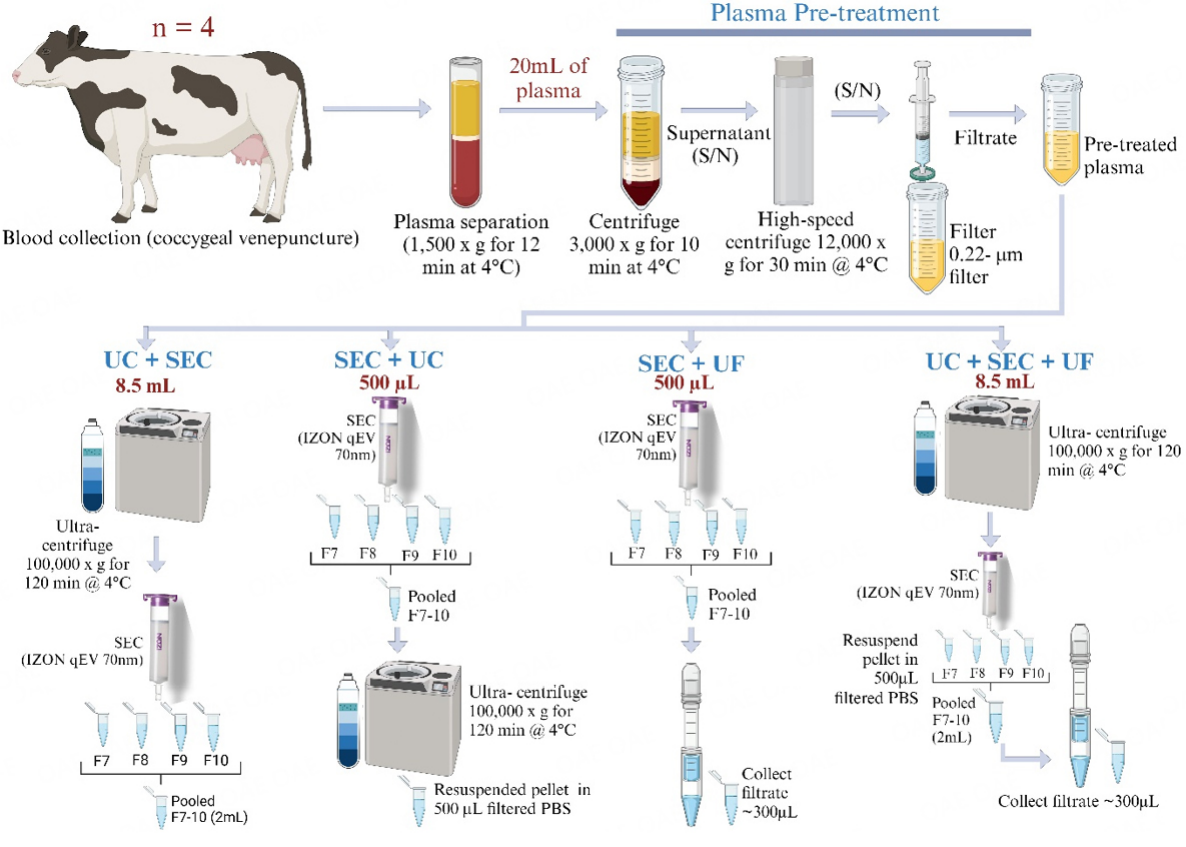
Schematic diagram of sEV isolation methods (Created with BioRender.com).

The above three sEV isolation techniques were combined in four different combinations to isolate the final sEV samples required for miRNA isolations. The schematic diagram of sEV isolation is in Figure 1.

(1) UC+SEC; (2) SEC+UC; (3) SEC+UF; (4) UC+SEC+UF.

### sEV characterization

The resulting samples, as described from the above four methodological approaches, were first quantified for the total protein content and subsequent experiments were conducted to determine exosome protein markers, particle size, shape, and concentrations [Supplementary File 1].

#### Total protein quantification (micro- Bicinchoninic acid assay)

Protein colorimetric detection and quantification was determined by micro BCA™ Protein Assay Kit (cat number 23235, Thermofisher Scientific, Brisbane, Australia). The microplate assay procedure was conducted with a linear range of 2-200 µg/mL and followed the manufacturer’s protocol as described previously^[30]^. Three technical replicates of bovine serum albumin (BSA) protein from each standard provided by the micro-BCA assay kit and two technical replicates of samples from each sEV isolation method were used to plate onto 96-well flat-bottom plate [Greiner CELLSTAR®, Sigma-Aldrich (Merck), Melbourne, Australia]. The samples and standards were solubilized in 1% w/v sodium deoxycholate (SDC) [Sigma-Aldrich (Merck), Melbourne, Australia]/deionized H_2_O, and the plate was incubated for 2 h at 37 °C and then absorbance measured at 562 nm using a plate reader (CLARIOstar Plus, BMG LABTECH, Melbourne, Australia).

#### Western blot

For characterization of exosome and non-exosome protein markers, three sEV biological replicates were pooled to create a representative sample, which was then utilized for three technical replicates (3 lanes in the Western Blot gel) for each of the four methodological approaches. Volumes required for WB were calculated according to the micro BCA total protein assay results to include a protein content of 10 µg in each sample. Samples were vacuum evaporated in a vacuum concentrator (cat number 5305000380, Eppendorf Concentrator plus, Sydney, Australia) and resuspended in 19.5 µL MilliQ water. The samples were processed as previously described^[21]^. Briefly, 4 × NuPAGE LDS sample buffer (NP0007, Thermofisher Scientific, Brisbane, Australia) and 10 × NuPAGE sample reducing agent (NP0004, Thermofisher Scientific, Brisbane, Australia) were added to the samples placed on ice to make up to a final concentration of 1 × and reduced for 10 min at 70 °C according to the manufacturer’s protocol. NuPAGE™ 4% to 12%, Bis-Tris, 1.0 mm, Mini Protein Gels, 10-well (NP0321BOX, Thermofisher Scientific, Brisbane, Australia) with Chameleon® Duo Pre-stained Protein Ladder (928-60000, Li-COR, Mulgrave, Australia) were used to resolve the proteins in the samples. For each gel, a plasma sample was used as the positive control as plasma is the source of sEV used in these experiments and milliQ water was used as the negative control. Polyvinylidene fluoride (PVDF) membrane (Bio-Rad Laboratories Pty Ltd., Sydney, Australia) was utilized to transfer the proteins from the gel and Trans-Blot® Turbo™ Transfer System (Bio-Rad Laboratories Pty Ltd., Sydney, Australia) was used for the transfer. Next, the PVDF membranes were blocked using a 1:1 solution of Odyssey Intercept blocking buffer (927-70001, Li-COR, Mulgrave, Australia) and phosphate buffered saline (PBS) [Sigma-Aldrich (Merck), Melbourne, Australia] for 1 h in a cold room (4 ºC). Then, the samples were incubated overnight in a cold room (4 ºC) (with agitation) with primary antibodies, recombinant anti-CD9 (1:250 dilution, Mouse monoclonal(NB500-494, Novus Biological, USA), recombinant anti-CD-81 (1:500 dilution, (NBP1-77039, Rabbit polyclonal (Novus Biological, USA), recombinant anti-flotillin-1 (1:500 dilution, Rabbit monoclonal (ab133497, Abcam, Melbourne, Australia) and recombinant-anti-BSA (1:5000 dilution, Rabbit polyclonal (ab192603, Abcam, Melbourne, Australia). The primary antibodies were diluted in a 1:1 diluted solution of Odyssey Blocking buffer and PBS; Tween-20 was added to make a 0.1% final concentration. On the second day of blotting, the PVDF membrane was washed four times in PBST (PBS + 0.1% Tween-20) for 5 min in each wash with agitation at room temperature (RT). Next, the membrane was incubated in the respective secondary antibody for 1 h in a cold room. Goat anti-Rabbit IgG (1:10,000 dilution, Li-COR, Mulgrave, Australia) was used for CD-81, FLOT-1 and BSA; Goat anti-Mouse IgG (1:10,000 dilution, Li-COR, Mulgrave, Australia) was used for CD-9. PVDF membranes were then again rinsed with PBST four times for 5 min in each wash. Finally, the membranes were imaged in a Li-COR Odyssey fluorescent scanner at 700 and 800 nm and were processed using Image Studio Lite v5.2 (Li-COR Biosciences, Lincoln, NE, USA). Plasma-derived sEV samples are expected to exhibit enrichment of EV markers (FLOT-1, CD9, CD81), while BSA, being an abundant plasma protein, is anticipated to be reduced in EV samples compared to unprocessed plasma.

#### Nanoparticle tracking analysis

The NanoSight NS500 instrument (NanoSight NTA 3.1 Build 3.1.46, Malvern Panalytical, Sydney, Australia) was used to determine the particle concentration, mean and mode sizes of sEV samples as previously described^[20,21]^. Statistical comparisons for NTA results (particle concentration, mean and mode sizes) were conducted in GraphPad Prism v9.5.0 utilizing a two-way analysis of variance (ANOVA) and multiple comparisons by isolation method, with significance set to *P* < 0.05. For NTA, three technical replicates from each methodological workflow that underwent sEV isolation process were utilized.

#### Transmission electron microscopy

Isolated sEV samples were imaged using a JEOL JEM-1400 TEM (JEOL, Sydney, Australia) operated at 100 kV, mounted with a 2K Teitz video and image processing systems (TVIPS) Charge Coupled Device (CCD) camera (TVIPS, Gauting, Germany) which was available in Central Analytical Facility (CARF, Queensland University of Technology, Brisbane, Australia). The samples were processed briefly according to the CARF guidelines which was previously published^[21]^. Briefly, 10 µL of a sample was mounted in formvar coated 200 mesh Copper (Cu) grids (ProSciTech, Townsville, Australia) for 1 min, and after blotting excess liquid, the Cu grid was mounted in 20 µL of 2% uranyl acetate (UA) for 3 min.

### MiRNA isolation and bioinformatics analysis

#### Optimized sEV miRNA isolation

**Figure 2:**
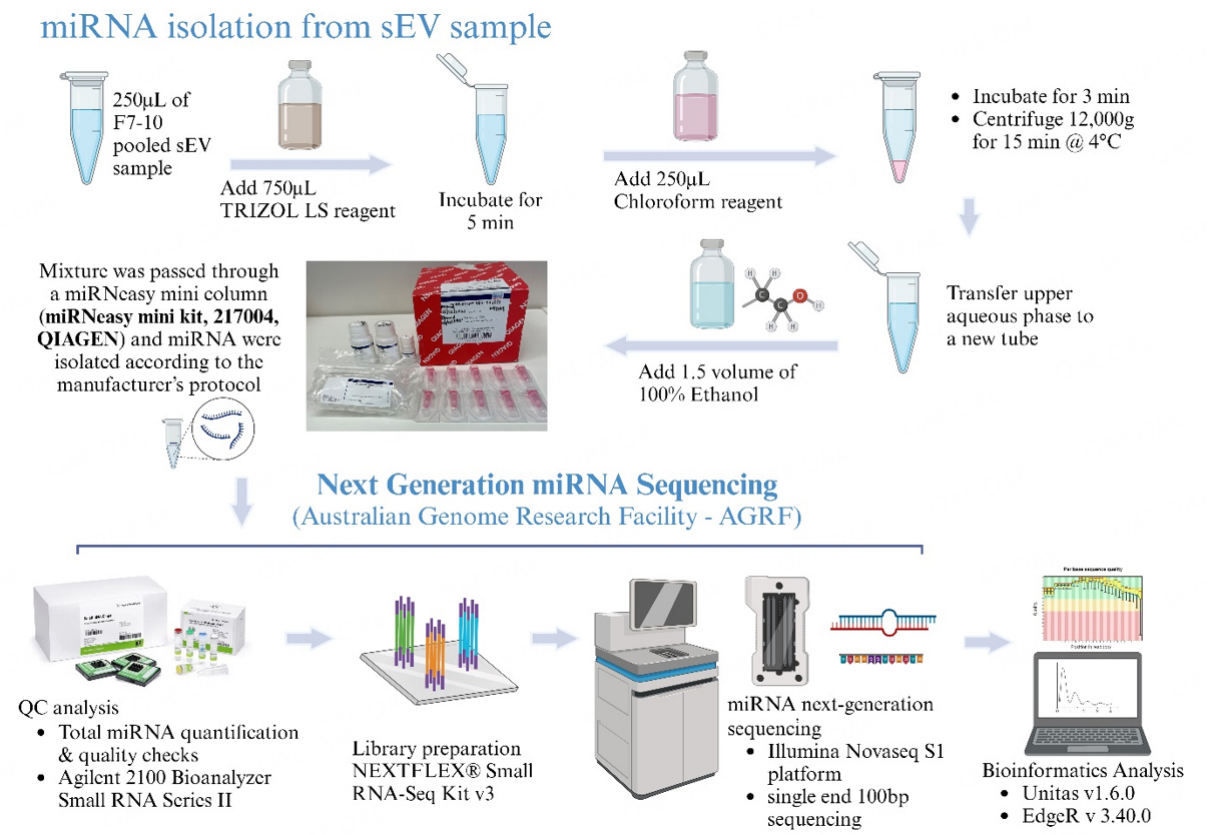
Schematic diagram of optimized miRNA isolation methodology (Created with BioRender.com).

MiRNA enriched from sEV samples isolated and characterized from all four methodological approaches through an optimized pipeline [Figure 2]. Rather than utilizing a total RNA sample for the next-generation sequencing, here we specifically purified a sample containing a majority of miRNA using the optimized miRNA isolating methodology. The input volumes of each sEV sample replicate were normalized by the total protein content; a volume of 25 µg total protein was used for each miRNA isolation.

SEV samples were thawed in ice and the experiments below were conducted in an RNase-free environment in a chemical fume hood. As the first step, total RNA digestion was conducted using TRIzol LS reagent (CAT#15596026; Invitrogen, Carlsbad, CA, USA) and was incubated with sEV samples (2.5 volume of sEV sample: 7.5 volume of TRIzol) for 5 min at room temperature (RT). Next, Chloroform (CAT#388306, Sigma-Aldrich (Merck), Melbourne, Australia) was added (2.5 volume of sample: 1.5 volume of Chloroform) for the phase separation of RNA from other impurities such as DNA, protein, *etc.*, and was incubated for 2 min at RT. Later, the samples were centrifuged at 12,000 × *g* for 15 min at RT and the transparent upper aqueous layer with RNA was carefully transferred to a new microcentrifuge tube. To improve the RNA binding capacity in next steps, a 1.5 × volume of 100% Ethanol was added to the recovered RNA sample. The mixture was then passed through miRNeasy mini spin columns (miRNeasy mini kit, 217004, QIAGEN) following the manufacturer’s protocol. Finally, RNase-free water supplied by the miRNeasy mini kit was passed through the column twice consecutively (each 40 µL) to collect 80 µL of sEV miRNA sample.

Four sample replicates of each isolation method were collected after the miRNA isolation step using miRNeasy columns, followed by next-generation sequencing (*n* = 4/isolation method). In preparing the control sample, two representative cow plasma samples were selected from each of the High Fertile and Low Fertile dairy cow groups. These samples were meticulously pooled before conducting miRNA isolation for the control sample, ensuring a comprehensive and robust representation. The control blood plasma miRNA sample adhered to the same miRNA isolation methodology (miRNeasy mini kit) and followed an identical downstream preparation and analysis pipeline as the remaining sEV miRNA samples.

#### MiRNA next-generation sequencing

Isolated and enriched sEV miRNA samples were sent to the Australian Genome Research Facility (AGRF; Melbourne, Australia) for primary quality control (QC) analysis and miRNA next-generation sequencing. Total miRNA concentrations for QC were conducted by Agilent 2100 Bioanalyzer Small RNA Series II (Agilent, Santa Clara, California, USA) to check the integrity and quality of enriched sEV miRNA samples.

Then, the library preparation of the samples was conducted using a NEXTFLEX® Small RNA-Seq Kit v3, and the next-generation sequencing was conducted using Novaseq S1 platform utilizing single-end 100bp sequencing. Each sample was conducted in triplicate on sequencing flowcell. Image analysis was performed in real time by the NovaSeq Control Software (NCS) v1.7.5 and Real-Time Analysis (RTA) v3.4.4, running on the instrument computer. RTA performs real-time base calling on the NovaSeq instrument computer. Then, the Illumina bcl2fastq2.20.0.422 pipeline was used to generate the sequence data. The resulting fastq.gz files were then downloaded from the AGRF data repository account and further processed for the bioinformatics analysis.

#### MiRNA bioinformatics pipeline

The bioinformatics pipeline including the codes is available in Supplementary File 2 and in the GitHub repository https://github.com/PevinduA/sEV-miRNA-annotation/tree/main. Briefly, the fastq.gz files were extracted and unpacked using WinZip® v27 (Mansfield, Connecticut, USA) package and the fastq files were used as the input to unitas v1.6.0 (https://sourceforge.net/projects/unitas/). The reads were also screened for the presence of any Illumina adapter/ overrepresented sequences (adapter sequence as per the library preparation kit: AGATCGGAAGAGCACACGTCTGAAC TCCAGTCAC) and the species was selected as *bos taurus*. The resultant bovine miRNA count tables from different sEV miRNA methodologies and the control bovine plasma sample were used in edgeR (version 3.40.0) of R package 4.2.2 (https://bioconductor.org/packages/release/bioc/html/edgeR.html) to perform differential expression (DE) of miRNAs between the groups. False discovery rate (FDR) analysis was performed to correct for multiple hypothesis testing and set to 0.05 (FDR < 0.05). Only miRNAs that met the FDR cut-off were considered statistically signiﬁcant. Graphical representation and statistical analysis were conducted using GraphPad Prism v9.5.0 utilizing a two-way analysis of variance (ANOVA) and multiple comparisons by isolation method, with significance set to *P* < 0.05.

#### Pathway analysis

Putative gene targets of DE miRNAs were identified using miRanda from miRNet version 2.0 (https://www.mirnet.ca/)^[31]^, TargetScanHuman 8.0 (http://www.targetscan.org/vert_80/)^[32]^ and miRWalk (http://mirwalk.umm.uni-heidelberg.de/)^[33]^. For miRNet, *bos taurus* was the selected organism, miRbase ID was chosen as the ID type and the target type was miRanda genes. For degree filter, “All network nodes” were selected with a cut-off value of greater than 3, greater than 100 cutoff was used for betweenness, and all networks were to connect with the shortest path^[34]^. For MiRWalk, putative target genes of 10 DE miRNAs were predicted with a cut-off binding probability > 0.98^[35]^. For TargetScanHuman 8.0, “cow” was selected as the species and each miRNA name was entered.

The Intersection of target genes from three miRNA target prediction tools were used as the confident target genes of DE sEV miRNAs. PANTHER v17.0^[36]^ was used to perform gene ontology (GO) enrichment analysis for the target genes of DE sEV miRNAs and ggplot2 v3.4.0 (https://ggplot2.tidyverse.org/) from R package 4.2.2^[37]^. Gene targets identified by miRNet were used to conduct GO compartment analysis.

To identify miRNAs with small extracellular vesicle (sEV) origin, we utilized miRanda through miRNet version 2.0 (https://www.mirnet.ca/) for the identification of miRNA gene targets. Subsequently, Panther GO cellular component enrichment analysis was conducted to specifically associate miRNAs with the "endosome (GO:0005768)" GO term, providing insights into the characterization of sEV-derived miRNAs.

## RESULTS

### NTA

**Figure 3:**
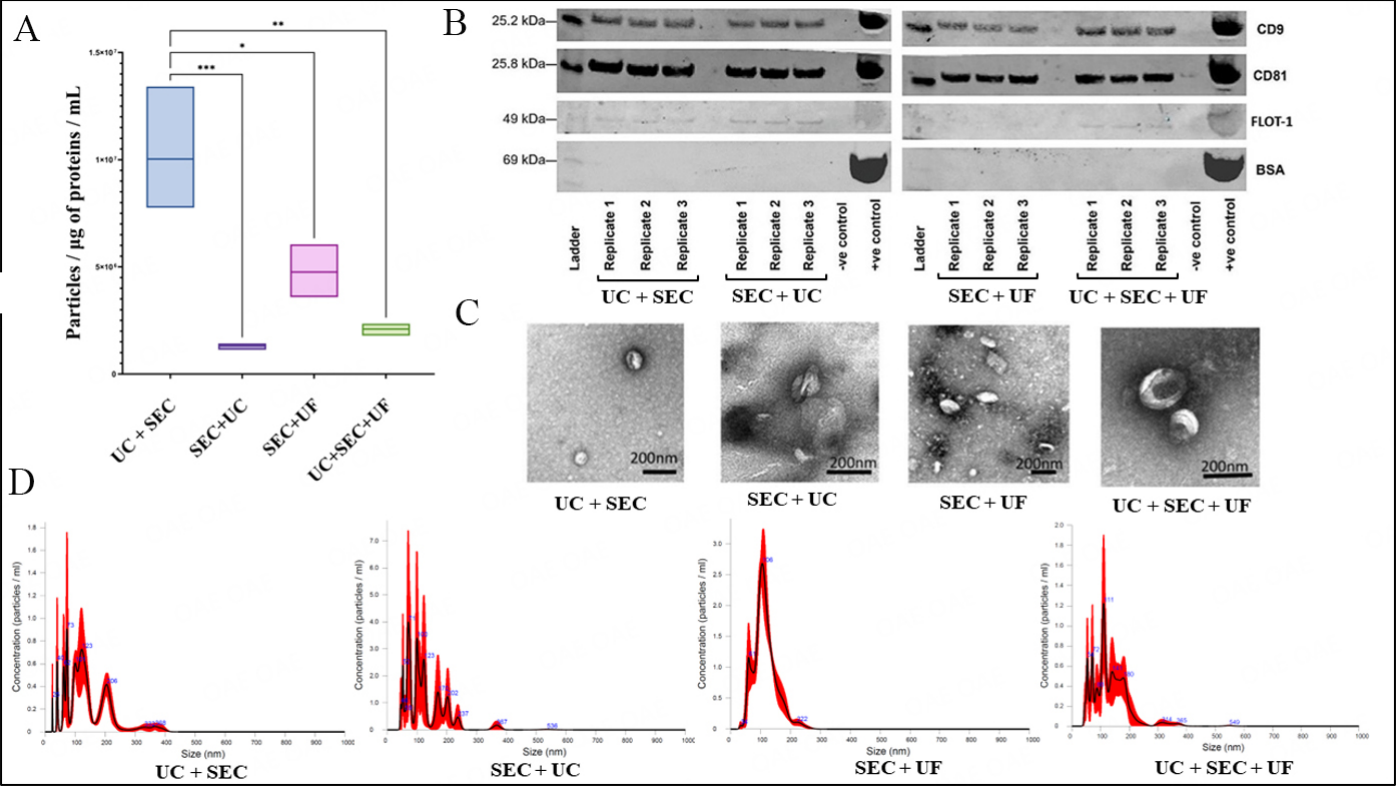
Characterization of sEV using NTA, western blot and TEM. (A) box plot (line at mean) of the number of particles per µg of total protein present per mL of sEV sample for the four different purification methods (*** *P* = 0.0008, **P = 0.0015, *P = 0.017). Three biological replicates were collected at the end of each isolation method for NTA; (B) relative abundance of exosome/EV membrane proteins (CD81, CD9, and Flot-1) and non-exosome protein marker (BSA) in sEV samples isolated from four different methodologies. Three biological replicates were pooled to create a representative sample, which was then utilized for three technical replicates (3 lanes in the Western Blot gel). Positive control is bovine plasma; (C) TEM images depict the spherical and cup-shaped sEV vesicles with appropriate size (~30-200 nm); (D) representation of original NTA images demonstrates that the EVs isolated from each method predominantly fall approximately between 30-200 nm in size.

The particle number normalized to micro-BCA total protein concentration depicts the presence of higher particle number in UC+SEC and the number of particles per µg per protein per mL yielded in UC+SEC is significantly higher than the rest of the three methods [Figure 3A]. SEC followed by UC (1.311 × 10^6^ particles/μg of proteins/mL) has fewer particles compared to SEC+UF (4.761 × 10^6^ particles/μg of proteins/mL), but the standard deviation in SEC+UF (1.231 × 10^6^ particles/μg of proteins/mL) is higher compared to SEC+UC (1.52 × 10^5^ particles/μg of proteins/mL). The additional filtration step in UC+SEC+UF (2.099 × 10^6^ particles/μg of proteins/mL) has compromised the particle number considerably, but the standard deviation is less than UC+SEC (2.831 × 10^5^ particles/μg of proteins/mL), indicating more consistency [Supplementary File 1].

The particle sizes of sEV isolated from all four methodologies fall into the defined particle size of sEV (30-200 nm). The most abundant particles (mode) were around 70-100 nm for all methods and the mean particle sizes varied around 110-140 nm size for all the sEV isolation methods. The particle size deviation among the replicates of SEC+UF is minimal compared to the rest and falls within sEV particle size (average mode size-103 nm; the average mean-115 nm) [Supplementary File 1].

### Western blot

Extracellular tetraspanin proteins CD81 and CD9 are present in sEV samples isolated using all the four methods. Exosome surface protein, Flot-1, was present but faint in SEV + UF sEV samples [Figure 3B]. However, bovine serum albumin (BSA) (negative sEV marker), which is highly abundant in plasma serum, was absent in sample replicates from all the methods and was only present in the positive blood plasma control sample. In the western blots, UC+SEC, SEC+UC, and UC+SEC+UF methods show the presence of three positive exosome/EV markers and the absence of a negative exosome/EV marker (BSA) which verifies sEV enrichment methodologies. The complete western blot images are attached in Supplementary File 1.

### TEM

The samples from all the four methodologies contained vesicles with sEV and exosome morphology, which were cup-shaped, spherical, and around 50-200 size diameter as observed under TEM [Figure 3C]. Complete uncropped images are available in Supplementary File 1. However, the extent of visible lipoproteins varied between sEV isolation methodological workflows; in particular, a minimal level of lipoproteins was observed in UC+SEC and UC+SEC+UF methods in contrast to SEC+UF and SEC+UC.

Collectively, the UC+SEC method produced a higher number of particles with the correct size and shape and the presence of tetraspanin CD9, CD81, and Flot-1 exosomal markers which validate those particles as sEVs [Figure 3]. Therefore, the UC+SEC isolation method is strategically successful in isolating sEV vesicle populations including exosomes. It is ideal for downstream applications which require higher quantities of validated sEVs.

### miRNA next-generation sequencing

**Figure 4:**
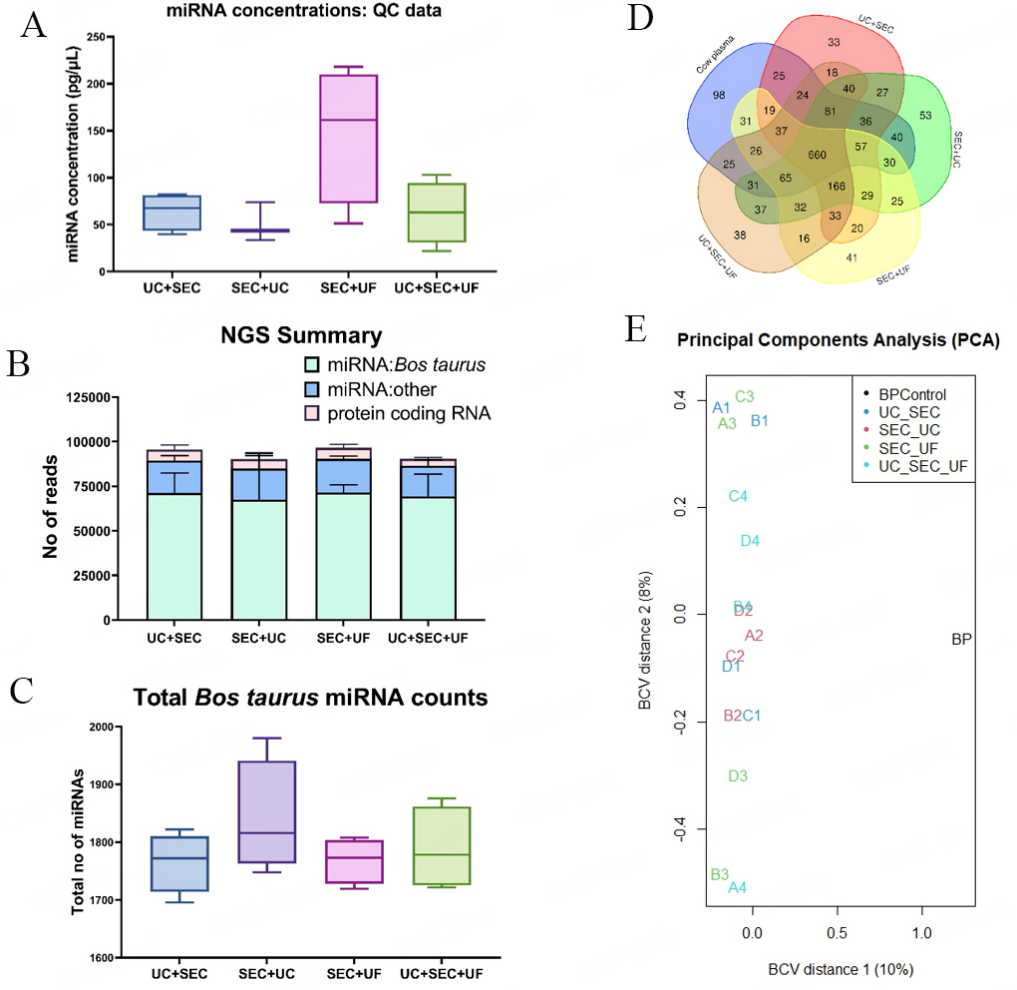
sEV miRNA characterization and next-generation sequencing demonstrated all four sEV miRNA isolation methodological approaches were successful (four technical replicates were utilized (*n* = 4) for each of the four isolation methods). (A) Quality control (QC) results of sEV miRNA samples quantified as pg/mL of miRNA concentration; (B) Summary of next-generation sequencing (NGS): different types of RNA read counts identified; (C) Total number of bovine (*Bos taurus*) miRNA counts identified; (D) the Venn diagram of shared and unique miRNAs in each method compared to the blood plasma control; (E) the principal components analysis (PCA) plot illustrates all sEV miRNA samples cluster together and away from the blood plasma-derived miRNA control. UC: Ultracentrifugation; SEC: Size exclusion chromatography; UF: Ultrafiltration.

The initial quality control (QC) data reveal that samples from all four sEV miRNA isolation methods include total miRNA average concentrations of more than 50 pg/µL [Figure 4A]. The single-end 100 bp next-generation sequencing discovered a majority of *Bos taurus* miRNA across all sEV isolation methodological approaches, confirming the efficacy of sEV miRNA isolation method used in this study: combination of TRIzol digestion and miRNEasy mini kit [Figure 4B]. The total list of miRNAs identified is available in Supplementary File 3 and the absolute miRNA counts for all the sample replicates are available in Supplementary File 4. According to miRbase v22, there are 2201 miRNAs in *Bos taurus* genome and more than 75% of them were present in all the sEV samples regardless of the sEV isolation method. SEC+UC resulted in a higher number of miRNAs but with a higher standard deviation as well. However, SEC+UF yielded an average of 1768 *Bos taurus* miRNAs with a lesser standard deviation [Figure 4C]. Quantitative data for the next-generation sequencing data are available in supplementary file 1. Interestingly, 660 miRNAs from all four sEV isolation methods were shared with cow plasma circulating miRNAs [Figure 4D]. Importantly, the principal components analysis (PCA) for miRNA results shows all the technical replicates from four methods clustered to the left side of the graph, demonstrating a clear separation from the control blood plasma circulating miRNA results [Figure 4E]. Therefore, it indicates a distinct set of miRNA cargo in sEV in contrast to the circulating miRNA present in the blood plasma, confirming the unique sEV miRNA cargo loading mechanisms.

### Identification of miRNA uniquely found in sEV

**Figure 5:**
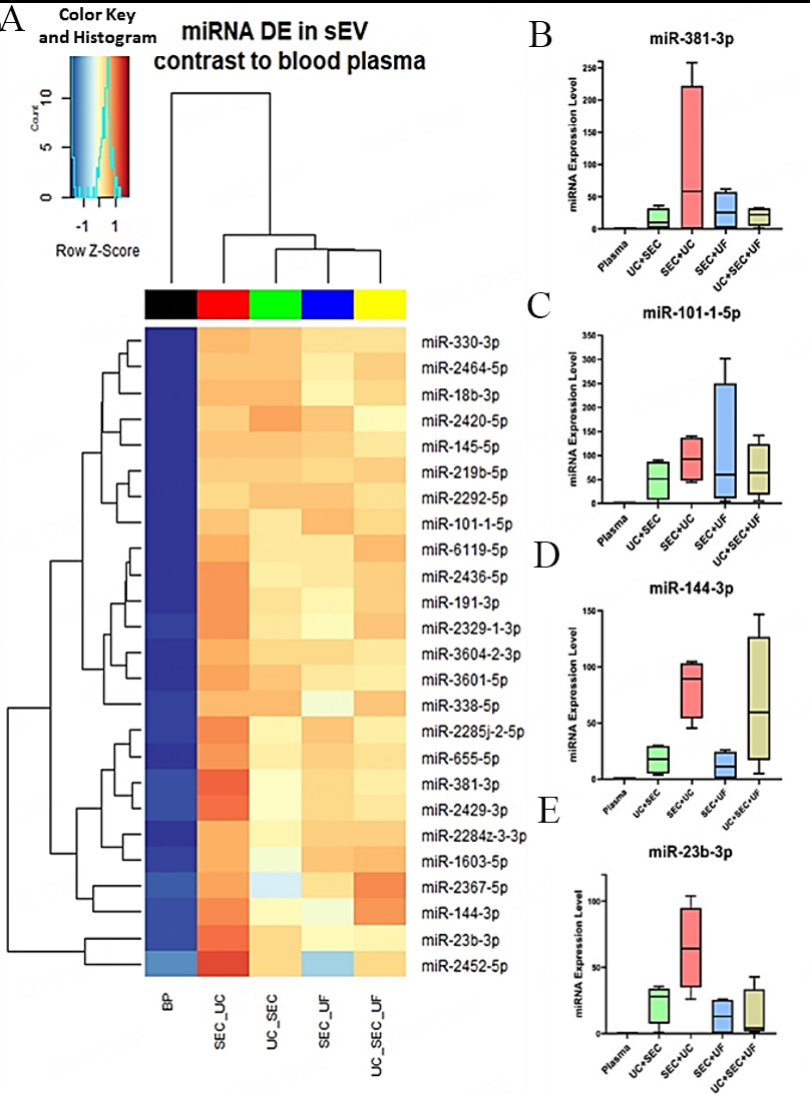
Unique miRNAs present in sEV compared to blood plasma. (A) Heatmap of DE miRNAs; Expression of miRNAs. Box and whiskers (minimum to maximum) plots of (B) miR-381-3p; (C) miR-101-1-5p; (D) miR-144-3p; (E) miR-23b-3p. BP: blood plasma; UC: ultracentrifugation; SEC: size-exclusion chromatography; UF: ultrafiltration; DE: differential expression.

Interestingly, our miRNA next-generation sequencing data revealed the presence of unique miRNA in sEV isolated from the above-mentioned methodological approaches compared to the blood plasma-derived miRNA profile [Figure 5A]. The differential clustering analysis revealed miRNA profiles from SEC+UF and UC+SEC+UF cluster together, likely due to the common final concentration step utilizing Amicon ultra-filtration devices.

Few of the miRNAs have already been found to be critical in biomarker diagnostics and therapeutics^[38,39]^. Comparatively, SEC+UC yielded higher miR-381-3p content [Figure 5B]; however, the rest of the methods identified the same miRNA as well. Expression levels of miRNA-101-1-5p in both UC+SEC and UC+SEC+UF were similar, although SEC+UF demonstrated higher dispersion of miRNA counts between the technical replicates [Figure 5C]. Counts of miR-144-3p were higher in UC+SEC+UF in contrast to UC+SEC, which suggests that the additional concentration step using ultra-filters enabled preferential isolation of this miRNA. However, the dispersion of count data between the technical replicates of UC+SEC was lower, and there was less variability in miR-144-3p isolation [Figure 5D]. MiR-23b-3p was also present in larger amounts in SEC+UC compared to the rest of the sEV methodologies, indicating purification using size-exclusion followed by the ultracentrifugation step preferentially isolated more of these two miRNAs [Figure 5E]. The average miRNA expression levels that were used for this analysis are available in Supplementary File 5.

Importantly, most of these 25 DE miRNAs in sEV samples have previously been identified to be specifically expressed in exosomes or EVs, and have been identified to have therapeutic value^[40]^ or as diagnostic biomarkers of various diseases^[38]^. The genes and proteins altered by DE miRNAs relate to extracellular vesicular origin and the complete gene ontology results are attached in Supplementary File 1.

### Identification of differentially expressed miRNA in blood plasma compared to sEV

**Figure 6:**
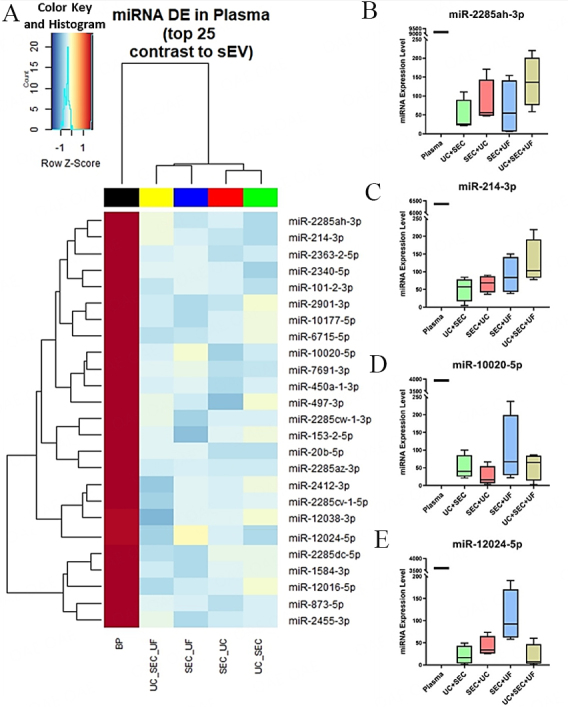
DE miRNAs present in blood plasma compared to sEV. (A) Heatmap of top 25 DE miRNAs. Expression of miRNAs. Box and whiskers (minimum to maximum) plots of (B) miR-2285ah-3p; (C) miR-214-3p; (D) miR-10020-5p; (E) miR-12024-5p. BP: Blood plasma; UC: Ultracentrifugation; SEC: Size-exclusion chromatography; UF: Ultrafiltration; DE: differential expression.

Most of the miRNA with low false discovery rates (FDR) were present at a higher concentration circulating in the blood plasma compared to the sEV samples. For the plasma-derived miRNAs that were present at a profoundly higher level, the sEV samples isolated from the four methods presented here also contained trace amounts of those miRNAs [Figure 6A]. Interestingly, UC+SEC+UF yielded a larger amount of miR-2285ah-3p [Figure 6B] and miR-214-3p [Figure 6C], while the SEC+UF method yielded sEV with higher amounts of miR-10020-5p [Figure 6D] and miR-12024-5p [Figure 6E]. This indicates that ultrafiltration concentration of samples preferentially isolates these two miRNAs. In summary, all four sEV isolation methodological approaches yielded satisfactory levels of miRNA content and are distinctly separated from the blood plasma circulating miRNA. However, UC+SEC sEV isolation stands out as the optimum method of sEV miRNA isolation in terms of a verified sEV subpopulation with a decent yield of miRNA.

### MiRNA target prediction and GO analysis for miRNA uniquely present in sEV

**Figure 7:**
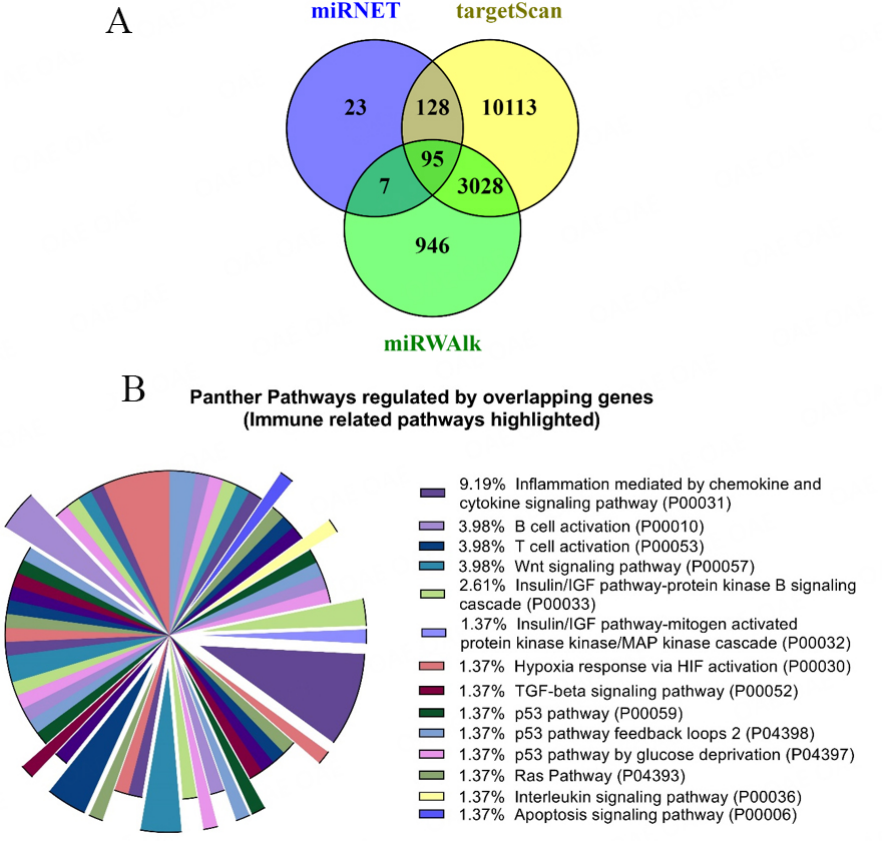
Gene target prediction and Gene Ontology (GO) enrichment analysis for miRNA uniquely present in sEV. (A) gene targets for 25 miRNA uniquely present in sEV were identified using miRNET, targetScan, and miRWalk miRNA target prediction tools and identified 95 shared/overlapping genes; (B) pathway analysis identified 95 genes that regulate mostly immune and inflammatory function (highlighted as exploded portions of the pie chart). This indicates 25 miRNA unique to sEV relates to immune function.

A total of 95 genes were identified as shared between the miRNA target prediction tool (miRNET, miRWalk, and targetScan) results [Figure 7A]. Interestingly, Panther GO analysis revealed that the 95 genes mostly regulate pathways related to immune and inflammatory function. Inflammation mediated by chemokine and cytokine signaling pathway (GO# P00031) represents 9.19%, which is the highest portion of the pathway analysis [Figure 7B and Supplementary File 6]. This may indicate a possible immune function of sEV through its uniquely packaged miRNA cargo.

Further, Panther protein class analysis identified that 95 genes regulate proteins related to extracellular matrix and cytoskeleton. It may indicate the domination of sEVs in the samples and the success of sEV isolation techniques [Supplementary File 1].

### The isolation methods result in different levels of cellular compartment enrichment

**Figure 8:**
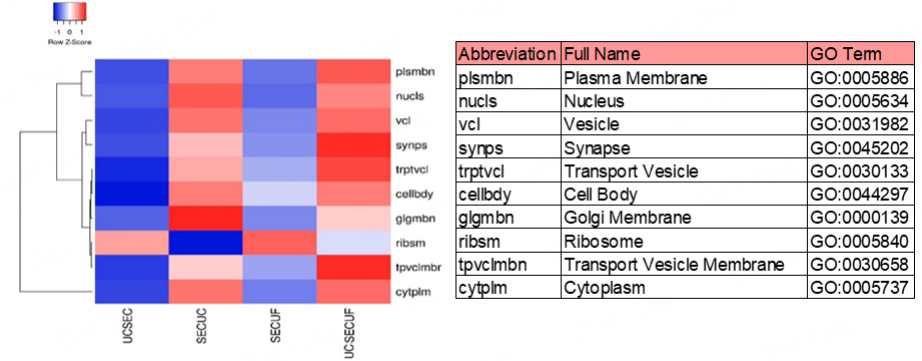
Comparative analysis of cellular component GO enrichment profiles for miRNA expression across four isolation methods.

In a broader context, Gene Ontology (GO) cellular compartment enrichment was conducted for miRNA profiles of each sEV isolation method. This analysis aims to investigate the varied expression levels of vesicle cellular compartments, providing a detailed comparison of enrichment patterns across different cellular compartments. GO terms for Vesicles (GO:0031982), Transport Vesicles (GO:0031982), and Transport Vesicle Membrane (GO:0030658) were comparatively enriched in the UC+SEC+UF method compared to the rest of the isolation methodologies. A higher enrichment of Golgi membrane (GO:0000139) and Ribosome (GO:0005840) was observed in SEC+UC and SEC+UF methods, respectively. However, a higher cytoplasmic cellular component was observed in both SEC+UF and UC+SEC+UF methods [Figure 8]. Furthermore, miRNA IDs related to “endosome (GO:0005768)" were identified in each isolation methodology and it indicates the endosome and/or exosome origin of those specific miRNAs. The full list of miRNAs with endosomal origin is available Supplementary File 1.

Therefore, the diversity in miRNA profiles among the four isolation methods did not exhibit significant alterations; distinct expression patterns of miRNAs were observed in terms of their function and origin.

## DISCUSSION

This study compared the efficacy of a combination of three sEV isolation methods for effective miRNA next-generation sequencing. The UC, SEC, and UF isolation methods were combined in different orders to form four methodological approaches and we demonstrated that all these methods can be utilized to isolate sEV miRNA with different yields and purity. The UC+SEC method enriched the largest number of sEV particles and characterization experiments verified that the particles were sEVs with expected size, shape, and exosomal protein markers. However, the SEC+UC method generated the largest number of miRNA IDs even with a lower sEV particle number. Including an additional UF step has compromised particle numbers; However, it was able to identify a greater quantity of miRNA IDs from the *Bos taurus* miRNA repository.

### The UC+SEC isolation yielded the highest sEV particle number, but with higher variability across technical replicates

Ultracentrifugation alone has been utilized as an EV isolation technique, and recently has been utilized to isolate EV samples with cellular origin required for miRNA qRT-PCR validations^[41]^. For UC, a 16 times larger starting volume of the blood plasma could be utilized in contrast to the SEC method; hence, the recovery of sEV particles from UC+SEC is essentially 10^3^ times higher in terms of number. Therefore, UC+SEC may be the ideal sEV isolation approach for pilot studies which aim to isolate sEV in large numbers for biomarker identification. The UC+SEC sEV samples contained all three Flot-1, CD9, and CD81 exosome protein markers which define EV size and shape, in contrast to the SEC+UF method, which isolated particles that do not express Flot-1. This protein marker expression validates the EV origin of miRNA from UC+SEC detected in the Agilent Bioanalyzer results. However, harvesting the sEV population from the UC pellet is a user-dependent procedure and thus the percentage recovery may vary during the manual reconstitution of the final 500 µL sEV sample. This variability is evident from the NTA particle numbers [Supplementary File 1] detected in the UC+SEC method compared to the rest of the three methodologies. Nevertheless, the UC+SEC samples generated more than 1,700 different *Bos taurus* miRNA IDs across technical replicates, confirming the capability of sEV miRNA detection from the isolated samples. A recent study combined UC+SEC isolation methods to isolate plasma EVs, but chose another combination of UC and SEC for miRNA validation using qRT-miRNA^[42]^. Isolation of higher yields of appropriate plasma sEV particles using UC+SEC combination encourages further miRNA isolation, which ensures the sEV origin of miRNAs. In another study, UC+SEC combination has previously been identified as the optimum sEV isolation method for protein enrichment, resulting in the highest protein IDs^[21]^.

### UF compromises the sEV yield after UC+SEC, but increases miRNA concentration

An extra purification step followed by UC+SEC using Amicon Ultra-2 filtration lowers the sEV particle number. However, UF decreases the variability of the sEV size distribution, in which the mode falls around 100nm of vesicular size. Interestingly, the miRNA concentration detected by the Agilent Bioanalyzer small RNA chip demonstrated a similar yet increased variability of miRNA concentration among four technical replicates of UC+SEC+UF in contrast to UC+SEC samples. Therefore, SEC+UF combination adds further value, as reported in a recent study utilizing UF alone which recovered the highest EV RNA yield compared to UC and SEC^[11]^. Adopting SEC+UF as the sEV isolation strategy may be beneficial as a time and labor-efficient methodology in contrast to UC-related sEV isolation strategies. Further, automated EV isolation technology based on magnetic beads has been introduced to replace UC^[43]^.

### Starting with SEC enables the detection of highest miRNA concentration per starting volume

The highest total number of miRNA IDs was identified in SEC+UC, although it was also the method that yielded the lowest total miRNA concentration. The SEC+UF method yielded the highest total miRNA concentration and detected a similar number of miRNA IDs as the UC+SEC method. Interestingly, the starting blood plasma volumes of both these methods were 16 times less than any of the methods that started with a UC step. In a separate proteomic-based EV isolation comparison study, Izon qEV 70 SEC columns were identified as the technique that isolated the most EV proteins from human blood plasma compared to other affinity-based sEV isolation techniques^[44]^. Nonetheless, SEC EVs have been shown to have higher functionality in terms of pERK/ERK ratios in an endothelial cell model^[12]^ and EV uptake in murine cardiac fibroblast cell model^[45]^ in contrast to EVs isolated using UC. Collectively, SEC enables the detection of sEV-derived miRNAs with a minimum sample volume using next-generation sequencing. Therefore, SEC coupled with either UC or UF could be useful for clinical studies which require the isolation of sEV miRNA from smaller volumes.

However, it is crucial to note that SEC+UC displays the highest dispersion, revealing a standard deviation (SD) of 98.85 to the mean of 1,840 (approximately 5%). In contrast, SEC+UF showcases the lowest variability in miRNA counts, with an SD of 39.27 to the mean of 1768.25 (roughly 2.2%). This disparity not only underscores the distinct characteristics of these isolation methods but also highlights the importance of carefully considering the sEV isolation sequence. The observation further demonstrates the bias on isolation techniques, emphasizing the need for thoughtful methodological selection in sEV studies.

### The sEV isolation methodology influences the expression levels of individual miRNA

Regardless of the combination of UC, SEC, or UF used, single-end 100 bp sequencing detected miRNA in all four methodological approaches, confirming the integrity of the sequencing platform and the miRNA bioinformatics pipeline. All samples passed the quality control Q30 test and yielded more than 20 million reads per sample. However, during the characterization of sEVs, the size, particle and presence of exosome marker distribution varied among the methods.

The diversity in miRNA profiles across the sEV isolation methodologies was not significantly altered. However, the chosen isolation methodology facilitated the purification of miRNA contents with varied expression levels among the four distinct sEV isolations [Figure 5B-E and Figure 6B-E]. Considering the distinct functional nature and cellular pathways influenced by each miRNA, the selection of the sEV miRNA isolation method becomes crucial, as it inevitably impacts the downstream applications of the isolated sEVs. This observation is confirmed again in Figure 8, where a differential expression of vesicle enrichment based on GO cellular compartment analysis was demonstrated across the four sEV isolation methodologies. An extra purification step of UF after UC+SEC enriched miRNAs related to EV cellular compartments [Vesicles (GO:0031982), Transport Vesicles (GO:0031982), and Transport Vesicle Membrane (GO:0030658)]. However, the UC+SEC+UF method enriched more nucleus, cytoplasm and ribosome cellular components in contrast to UC+SEC. The influence of sEV isolation methodology on miRNA expression is similar to our parallel study on proteomic content, where aliquots of exact samples used in this study were utilized^[21]^. Interestingly, CD63 protein was not detected in any of the sEV samples isolated through the four isolation methodologies. The absence of CD63 is intriguing, especially considering the higher abundance of 24 EV-specific proteins, including tetraspanin CD9 and CD81, each with a confidence score exceeding 99%, confirming the EV characteristics in our samples. This tetraspanin distribution pattern may be attributed to the selected small extracellular vesicle (sEV) isolation methods used in this comparative study and the specific type of samples we utilized, i.e., bovine plasma samples. Therefore, it hinders the incorporation of CD63 exosome marker for characterization in this study; the orthogonal characterization techniques used (NTA and WB) confirm the presence of particles that are both within the widely established size range of small EVs and positive for known EV markers FLOT-1, CD81, and CD9.

### Identifying miRNAs uniquely expressed in sEV as potential exosome miRNA markers in contrast to circulating miRNAs

Mir-381-3p has been identified recently as an exosome miRNA diagnostic biomarker for human colorectal cancer (CRC). Specifically, it was highly abundant in the blood serum exosomes of healthy controls compared to the CRC patients^[38]^. Exosome-derived miR-381-3p has also been identified as a cancer therapeutics, demonstrating anti-cancer properties^[40,46]^, and has shown successful delivery to cells via exosome loading^[39]^. Furthermore, the presence of miR-101^[47]^, miR-23-3p^[48]^, miR-144-3p^[49,50]^, miR-18b-3p^[51,52]^, and miR-330-3p^[53]^ in exosomes or EVs has previously been validated as potential diagnostic biomarkers or for exosome therapeutics.

During exosome biogenesis, RNA molecules including miRNA are selectively packaged into sEV^[54]^, and many studies have reported that the EV RNA profiles differ profoundly from those of their cells of origin^[55]^. The vesicular membrane protects many unstable small RNA species^[56,57]^ and usually inhibits their activity on target RNA sequences via 3’UTR direct binding^[58,59]^. Transforming growth factor-beta-3 (TGFβ3) mRNA’s 3’UTR site is a direct target of miR-381-3p^[46]^. Therefore, the absence of miR-381-3p in the blood plasma miRNA profile [Figure 5A and B] may be influenced by the abundance of TGFβ3 mRNA in the blood plasma^[60,61]^.

Further, more than 50 miRNAs were identified in each sEV miRNA isolation methodology which has sEV origin and specifically targets genes related to endosome (GO:0005768) cell compartment enrichment [Supplementary File 1]. This analysis revealed that the majority of miRNA IDs were shared^[33]^ between all four methodologies; however, SEC+UC method resulted in the highest yield^[56]^ and unique^[5]^ miRNA IDs with sEV origin relative to the rest of the three methods.

### Uniquely expressed miRNA in sEV indicates a possible immune function

The functions of sEV are heterogeneous; however, our results indicate that miRNAs only present in the sEV compared to circulating blood plasma may regulate the immune function. Specifically, they appear to predominantly affect inflammatory signaling pathways that can directly compromise immunity. Therefore, sEV and its miRNA cargo can further be utilized for immune-related diagnostics and/or therapeutics.

### The level of Lipoprotein contamination may be reduced in sEVs isolated by UC+SEC and UC+SEC+UF

According to TEM images, sEV isolations started with UC have lesser lipoprotein contamination in contrast to SEC+UC and SEC+UF, which contained higher lipoprotein abundance. It confirms that the lipoprotein particles share similar biological properties including size and shape, as has previously been documented^[62,63]^. sEV studies are being conducted using alternative sEV isolation methods to separate sEV from lipoproteins, such as density gradient centrifugation^[64]^, magnetic bead-based methods^[65]^, and acidification^[66]^; however, it will be worthwhile to investigate if those sEV isolation methods preserve the miRNA integrity. Therefore, we recommend future studies to investigate whether miRNA is exclusively present in sEVs and/or if it co-localizes with lipoprotein particles.

In contrast, miRNAs that exhibit high expression in blood plasma with a higher confidence level (low FDR value and higher fold-change) are those that were minimally expressed and/or absent in sEVs [Figure 6A].

## CONCLUSION

We herein present four sEV isolation methodological approaches for miRNA next-generation sequencing and the unique presence of immune- and inflammatory-related miRNA cargo packaged in sEV. Given the higher tendency of sEV and its miRNA cargo to be utilized for diagnostics and therapeutics in immunology, successful sEV miRNA isolation methods with higher purity are essential. Thus, this study elucidates that four combinations of UC, SEC, and UF passive sEV isolation methodological approaches enable successful miRNA isolation and their characterization using next-generation sequencing. In summary, the UC+SEC combinatory method produced the highest yield with correct mode and mean sizes of sEV, optimum sEV miRNA yield, and optimum *Bos taurus* miRNA IDs which demonstrated a clear separation from circulating plasma miRNAs. Although SEC+UC identified the highest *Bos taurus* miRNA IDs, the yields of sEV and miRNA were the lowest. The highest sEV miRNA yield was generated by SEC+UF. Importantly, we identified unique miRNAs that could be used as miRNA markers to identify and characterize exosomes and sEV.

Therefore, the choice of sEV enrichment and miRNA isolation strategy may provide different results in the research setting and for downstream miRNA characterization techniques. Interestingly, selection of an optimal sEV isolation method may depend on the required downstream analyses of biomolecules, but our findings could translate to different directions of analysis as well. In a separate study, we have shown that the same sEV isolation combinations could be used for sEV proteomic analysis and sEVs isolated through UC+SEC combination yielded the highest protein IDs^[21]^. We were able to identify unique miRNAs in sEV and blood plasma, which mostly regulate immune and inflammatory pathways. We suggest that further validation experiments be performed to establish these miRNAs as positive or negative exosome markers. Furthermore, we suggest conducting validation experiments using miRNA qRT-PCR assays and bioinformatic analysis to investigate whether the miRNAs generated are genuinely from sEV origin, and not from circulating protein-bound miRNAs^[67]^. However, it is noteworthy that the sequence of sEV isolation not only introduces a potential bias in the size and type of the isolated sEV, but also influences the sEV miRNA content and their functions, as demonstrated in this study results.

Failing to adopt the optimum sEV isolation method and miRNA isolation strategy will exhaust resources and hamper experimental validity. Therefore, our work provides guidance to navigate blood plasma sEV-derived miRNA isolation methodologies essential for next-generation sequencing.

## DECLARATIONS

### Availability of data and materials

All the data are available online in Replication data for sEV miRNA isolation method comparison^[68]^.

### Copyright

© The Author(s) 2024.
