# Supplementary material for: A comparative analysis of small extracellular vesicle (sEV) micro-RNA (miRNA) isolation and sequencing procedures in blood plasma samples
Source: Extracell Vesicles Circ Nucl Acids. 2024 Feb 29;5(1):119–37. doi: 10.20517/evcna.2023.55 (PMC11648519; doi:10.20517/evcna.2023.55)
Supplement: Supplementary file 1 [file evcna-5-1-119-SupplementaryMaterials.zip › evcna5055-SupplementaryMaterials/Supplementary File 1.docx]

Supplementary Figure 1: Western Blot


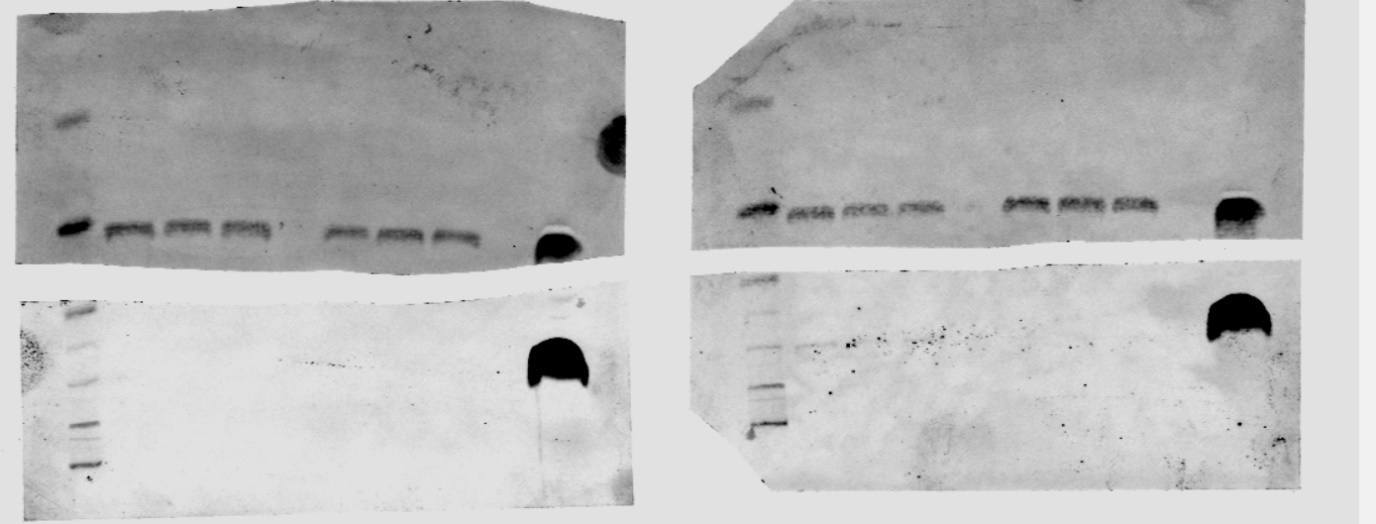

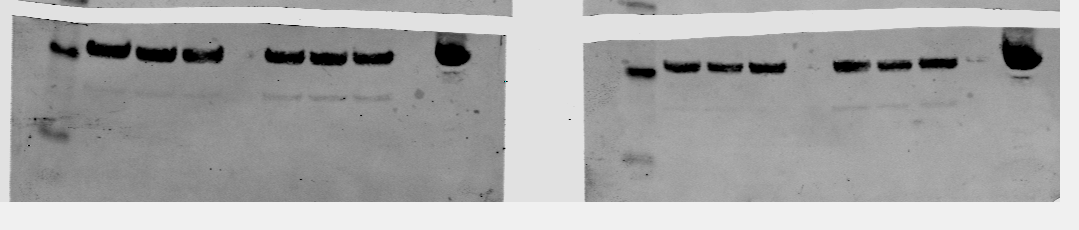


**UC + SEV + UF**R1 R2 R3

**UC + SEV**
R1 R2 R3

**SEV + UC**
R1 R2 R3

**SEV + UF**
R1 R2 R3

+ve control

-ve control

+ve control

-ve control

CD9
25.2 kDa

CD81
25.8 kDa


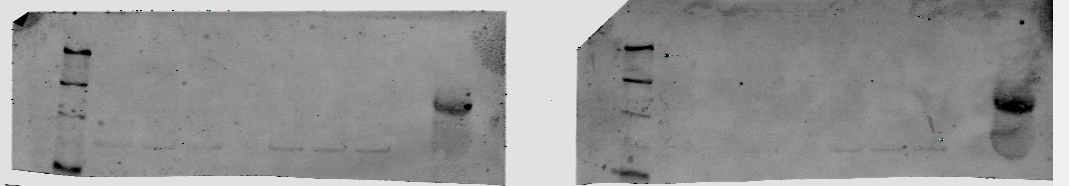


Flot-1
49 kDa


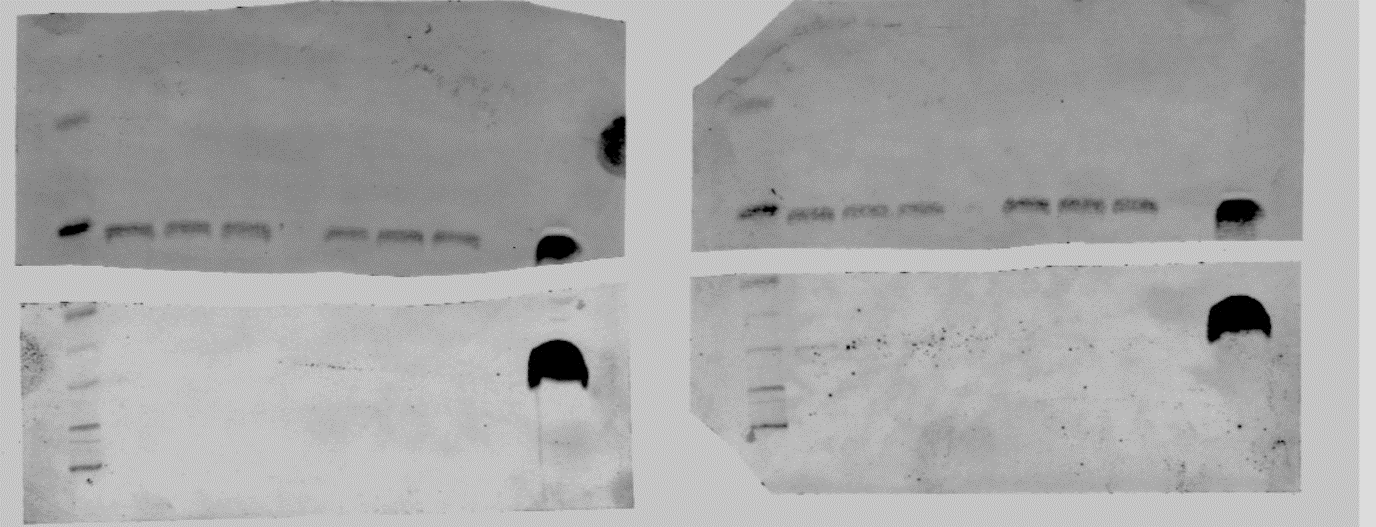


BSA
69 kDa

Supplementary Figure 2: TEM


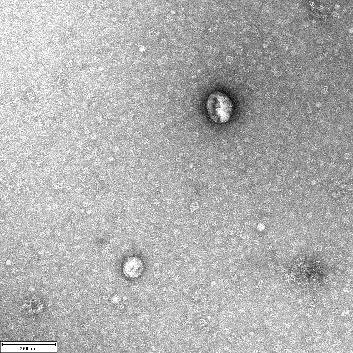


UC + SEC


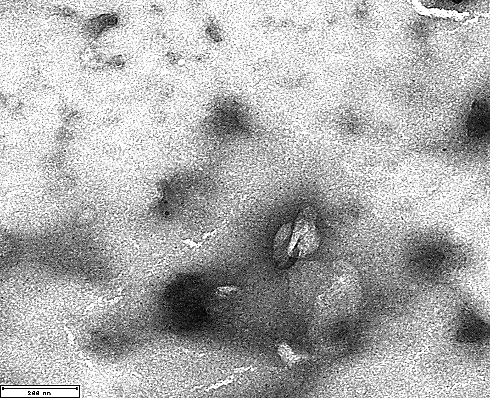


SEC + UC


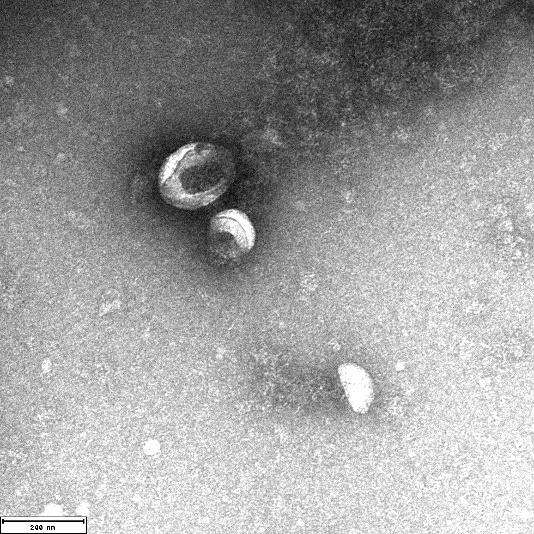


UC + SEC + UF


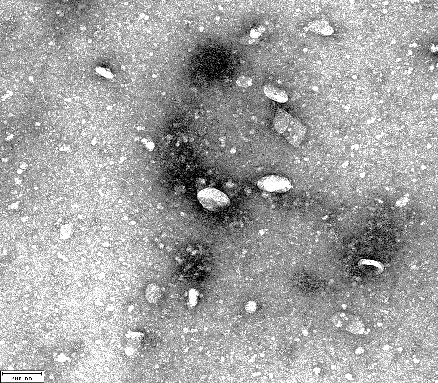


SEC + UF

Supplementary Figure 3: NTA


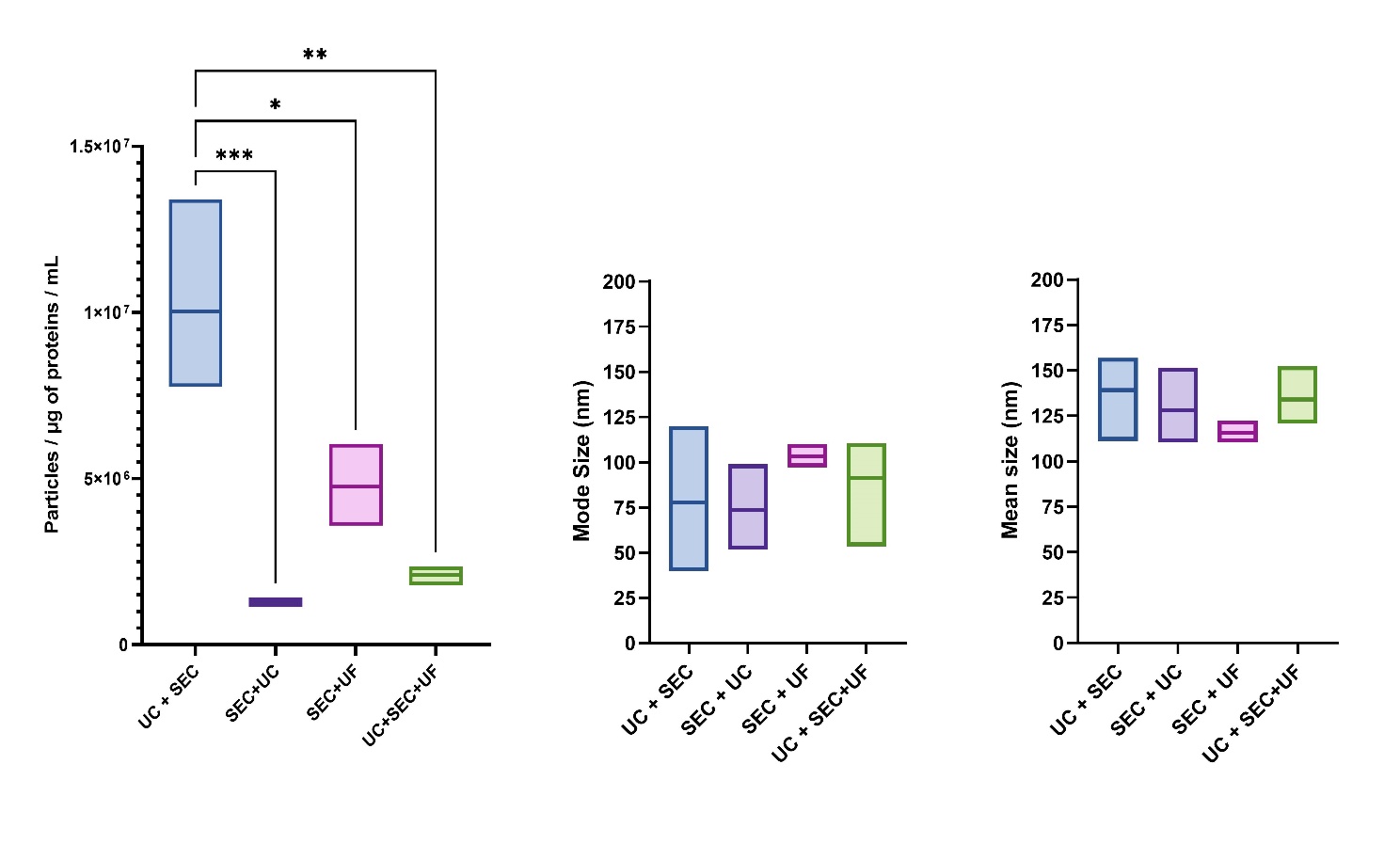


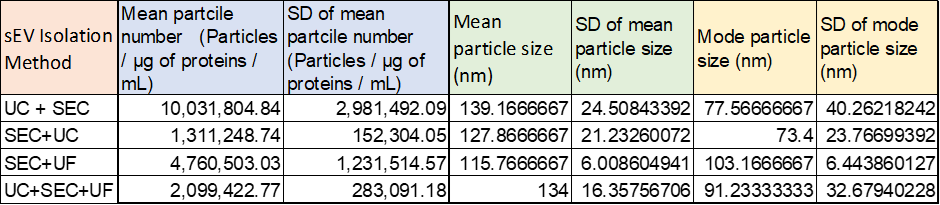


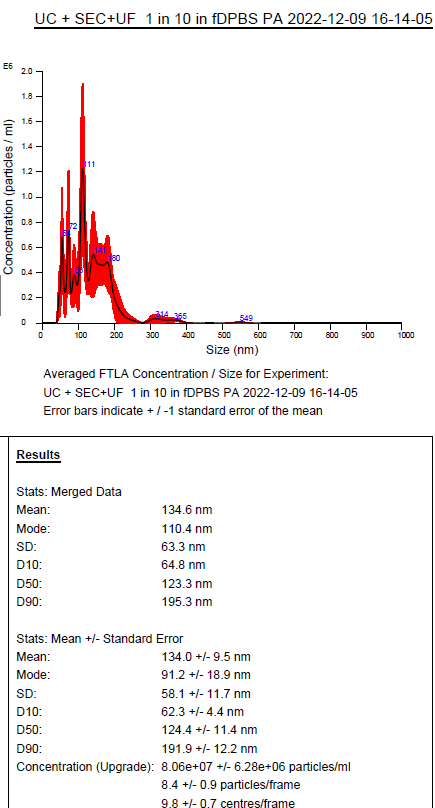

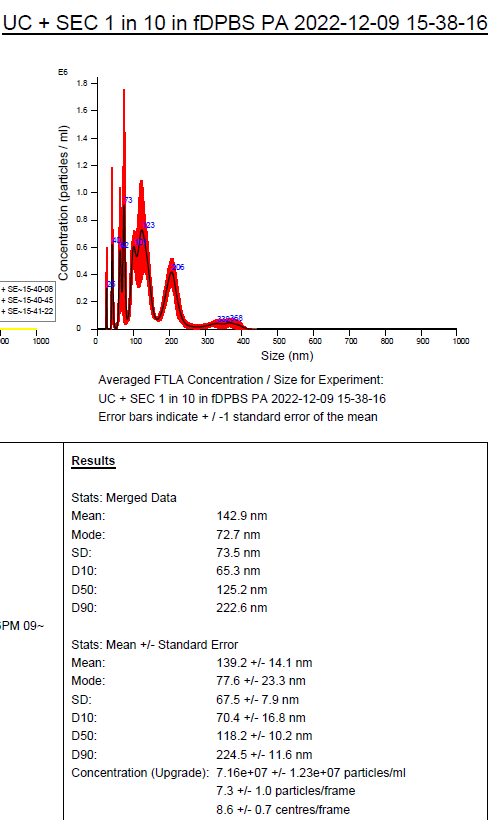


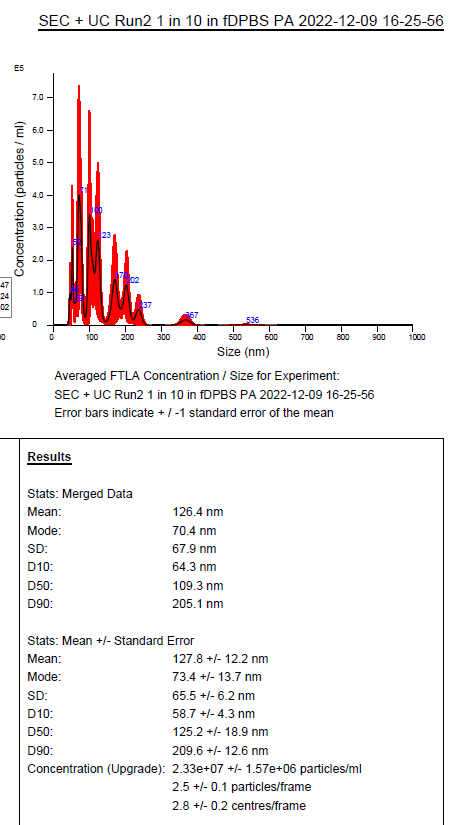

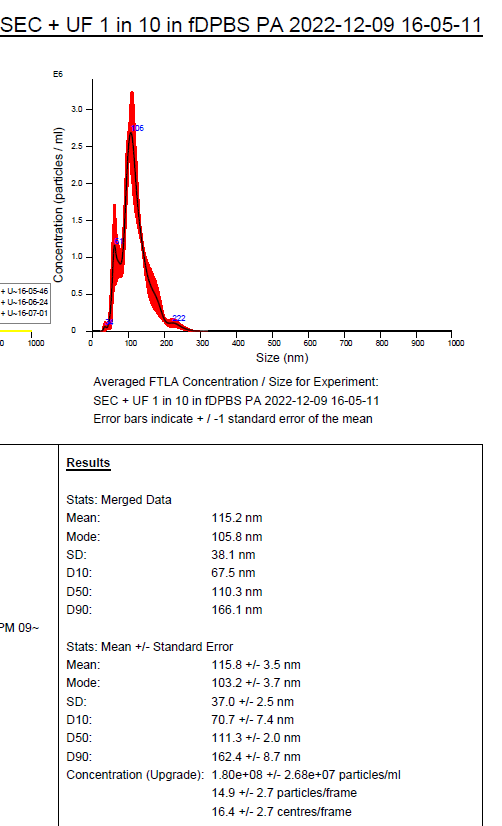


Supplementary Figure 4a: GO enrichment of UC+SEC sEV miRNA profiles


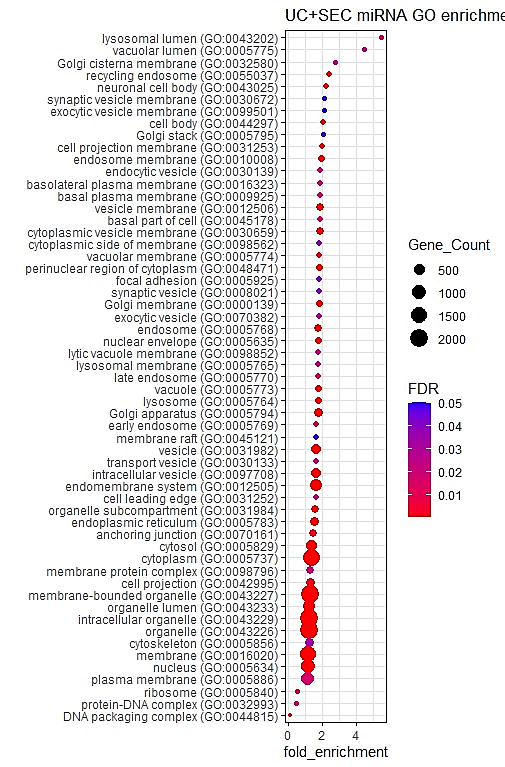

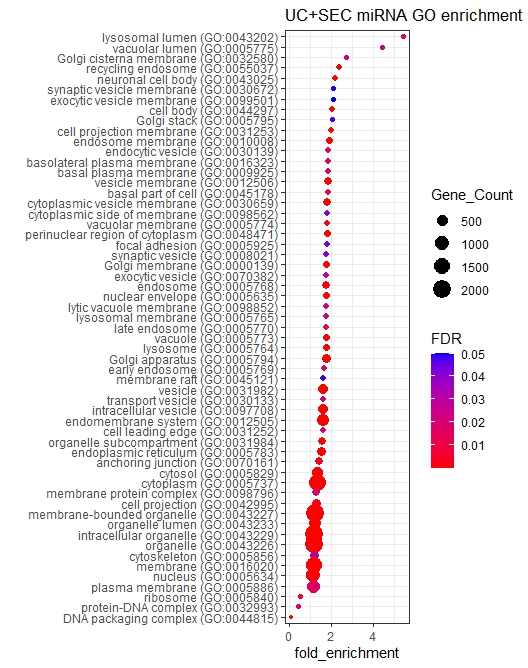


Supplementary Figure 4b: GO enrichment of 25 miRNAs expressed only in sEV samples.


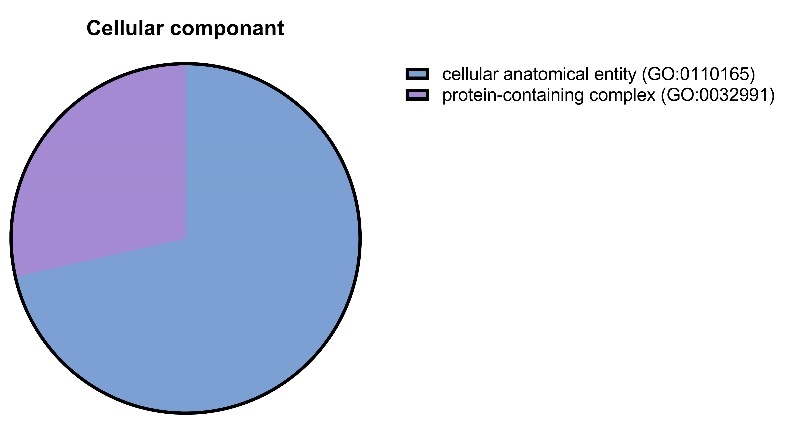

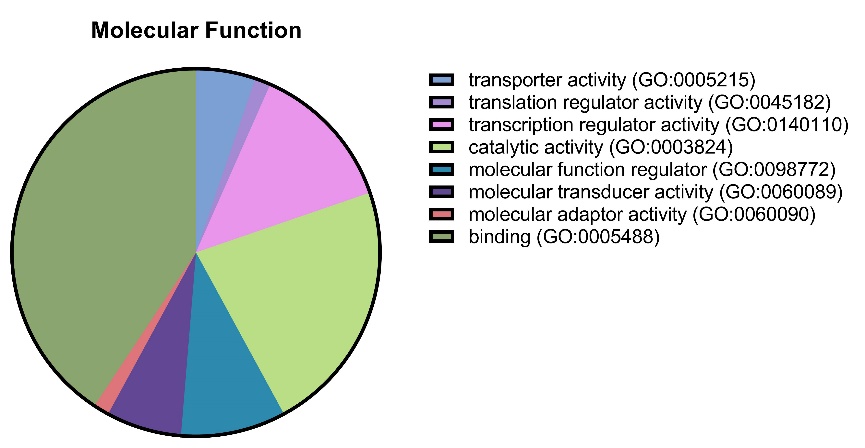

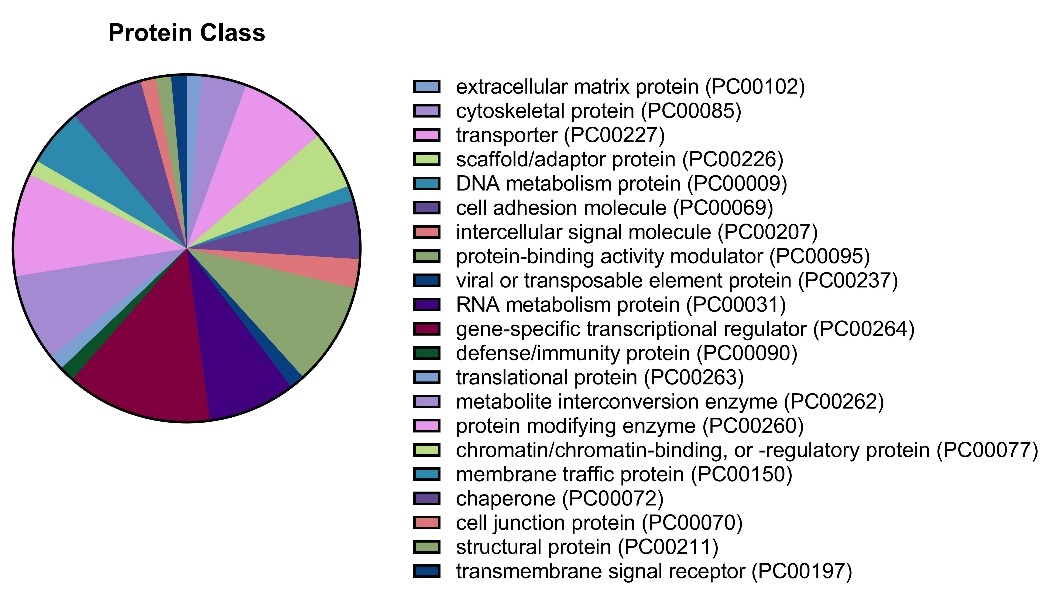

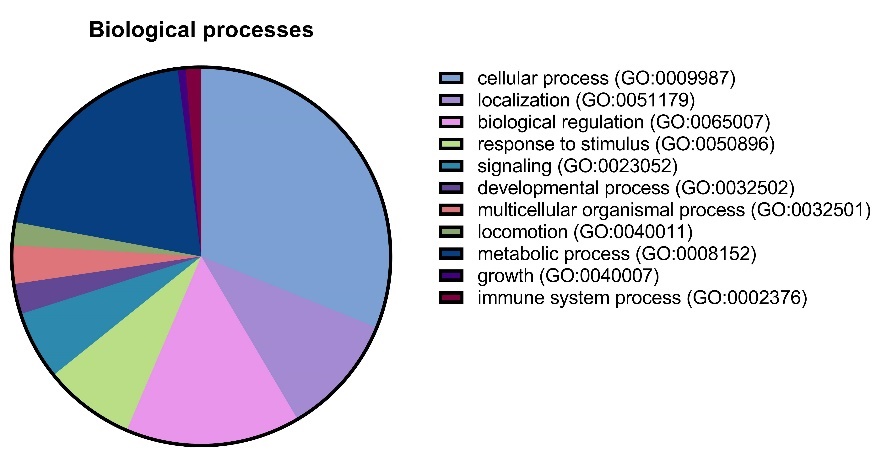


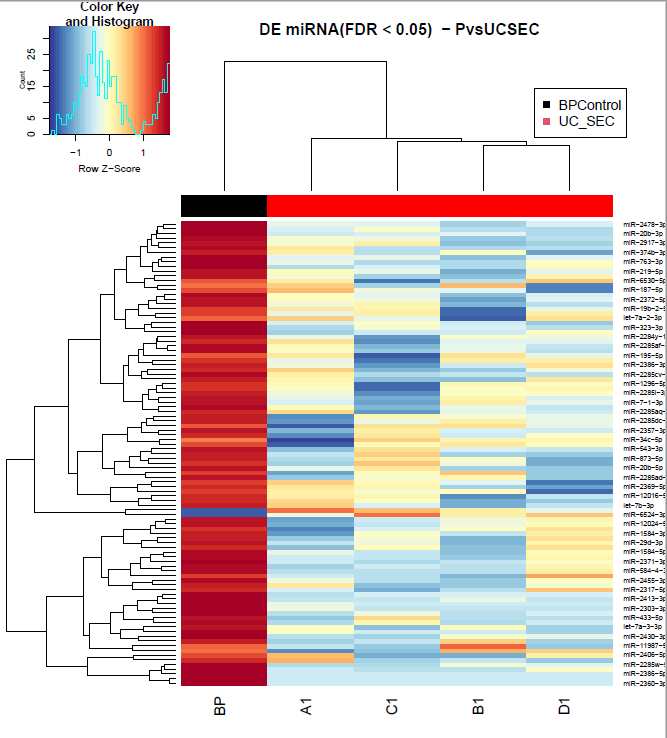
Supplementary Figure 5a-d: Differential expression of miRNA between plasma and sEV isolation methodologies. sEV samples from four methods individually shows a separation from plasma circulating miRNA profile.

Fig 5a


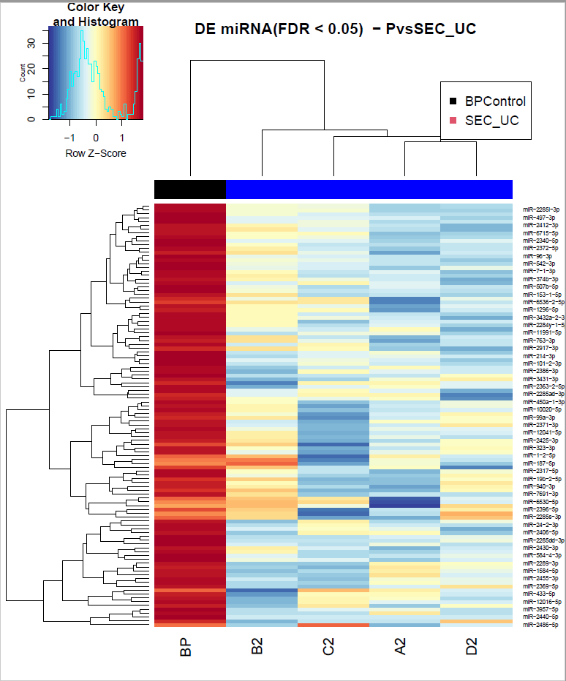


Fig 5b

Fig 5c


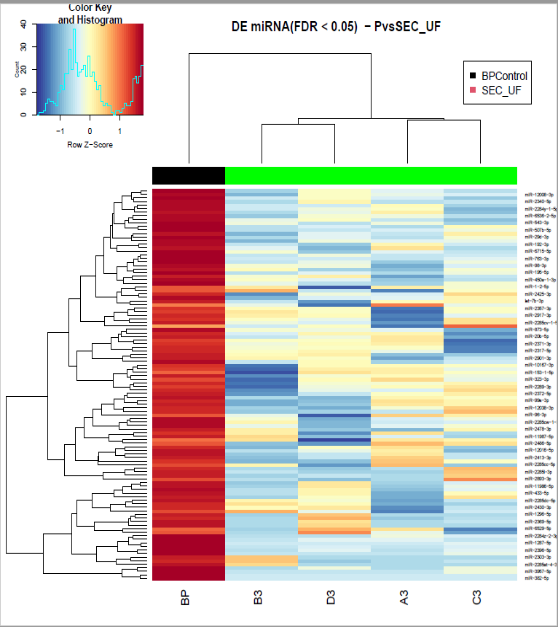


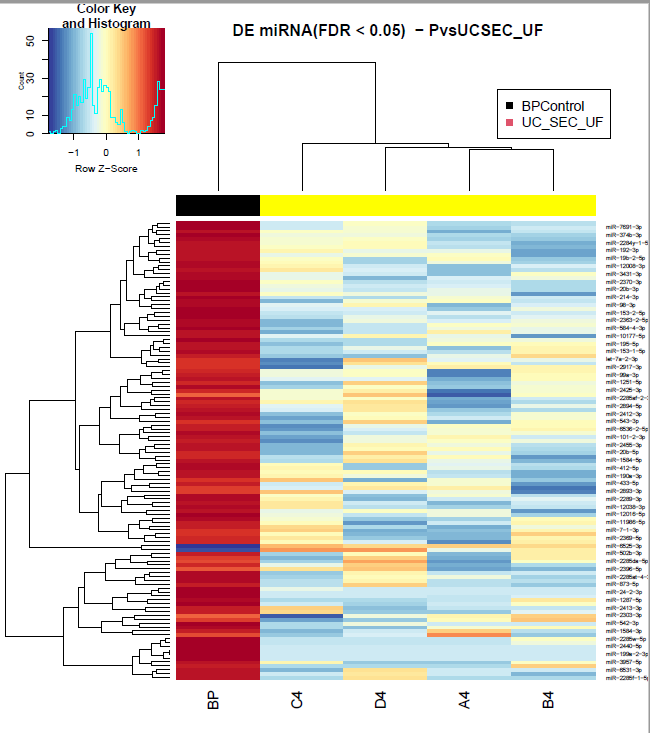


Fig 5d

**Quantitative next-generation sequencing data**

**Table 1: miRNA concentrations: QC data**

**Table 2: Total No of miRNAs identified.**

**Table 3: Next-generation sequencing summary**

**Table 4: Total RNA abundance per starting volume of the sample**

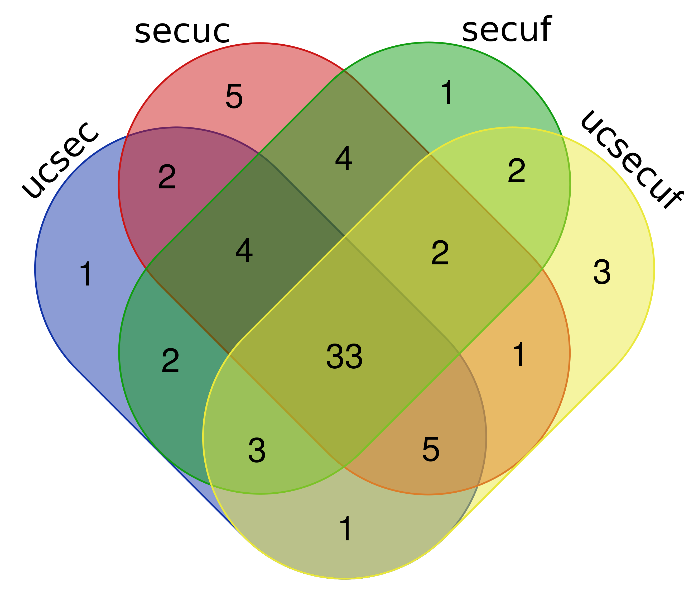
**Table 5: miRNA associated with sEV (miRNA enriched for the 'endosome (GO:0005768)' GO term.**


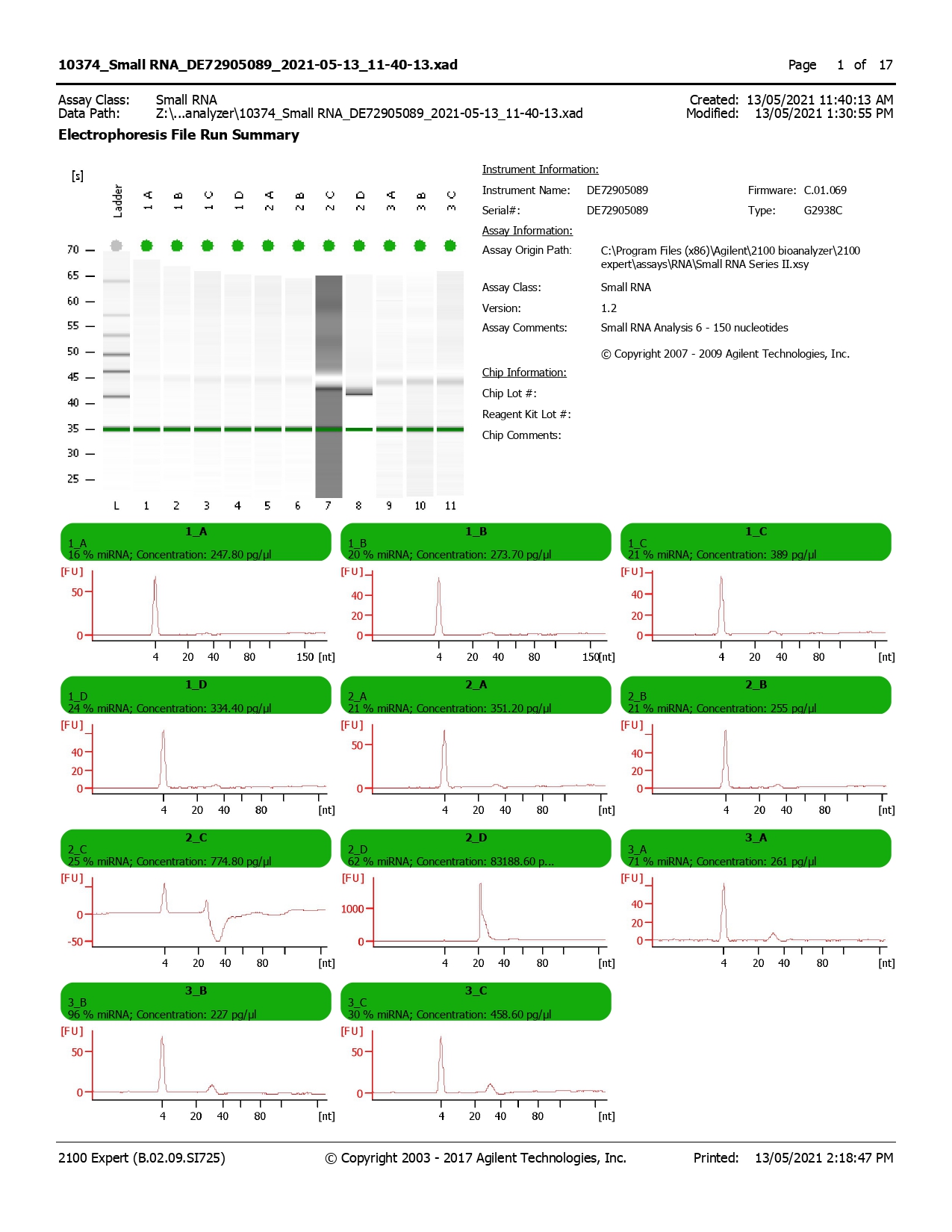
**Figure 6: Original QC results**

**File 1 (page 1-17)**


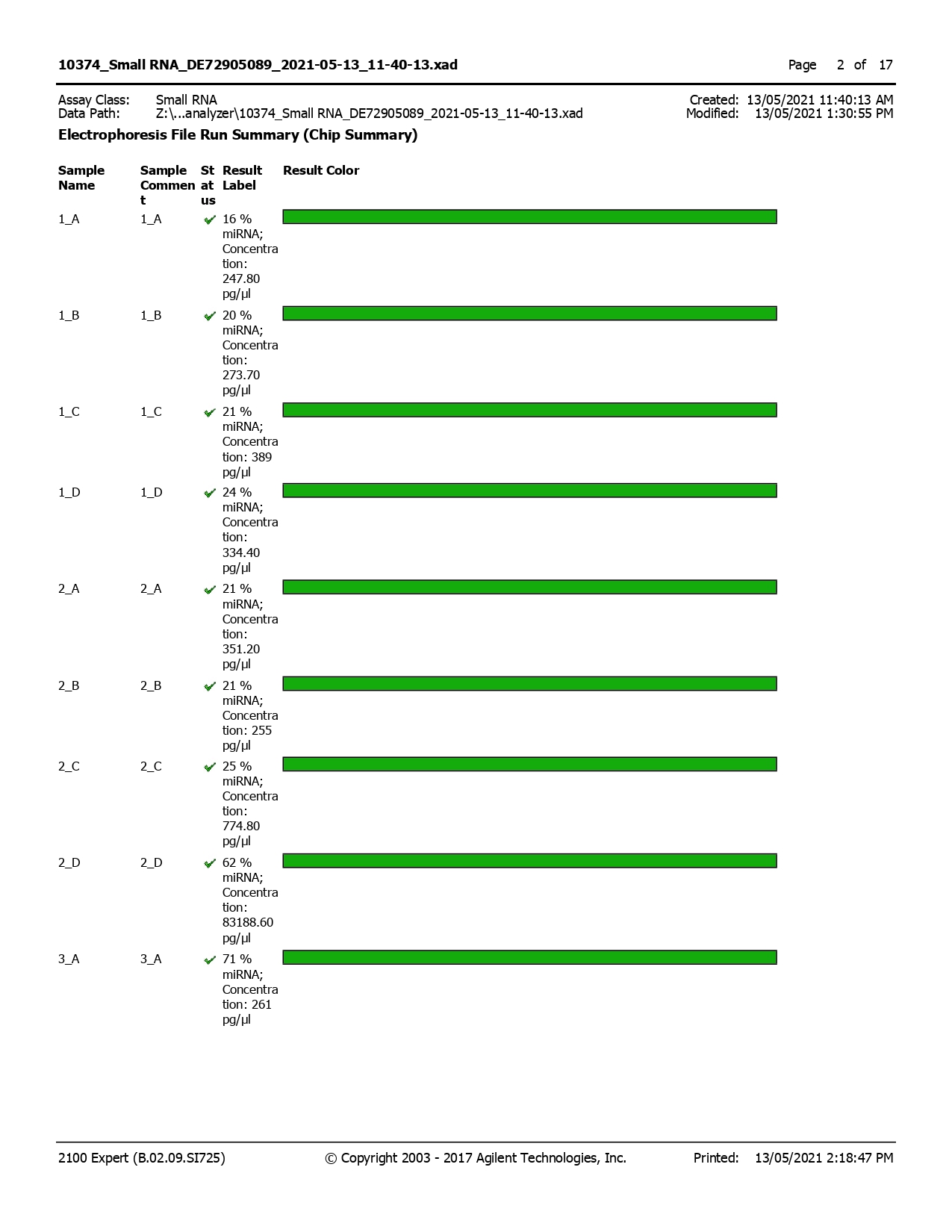

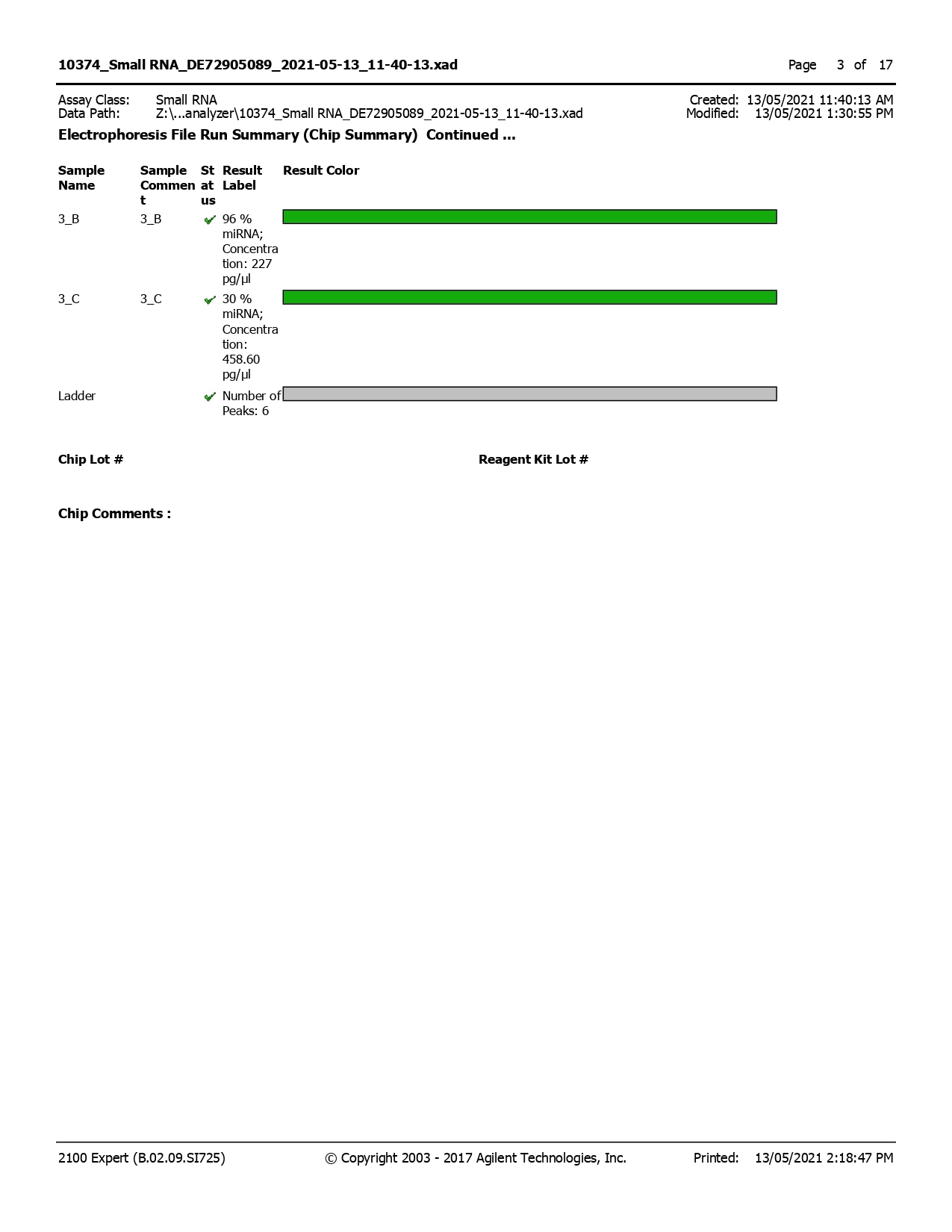

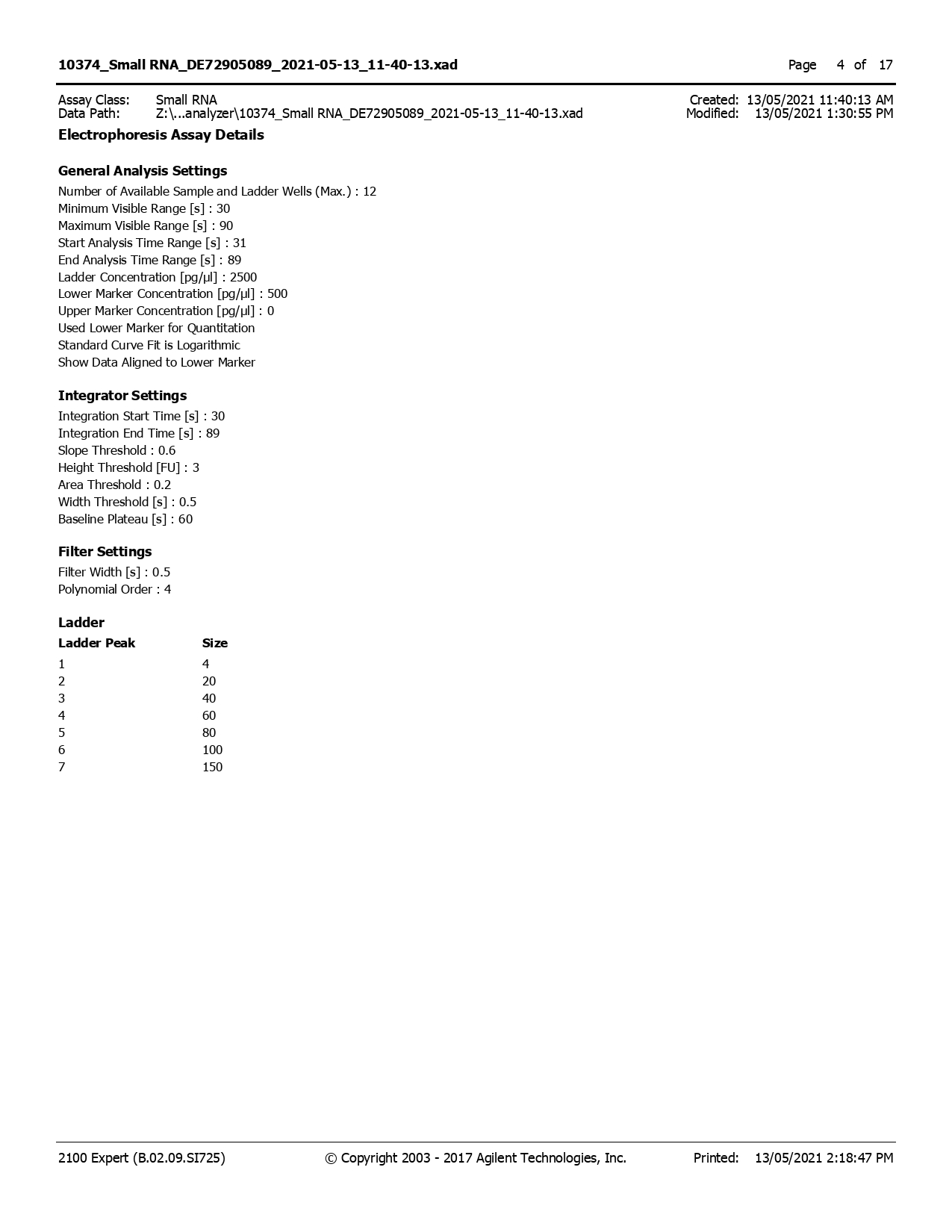

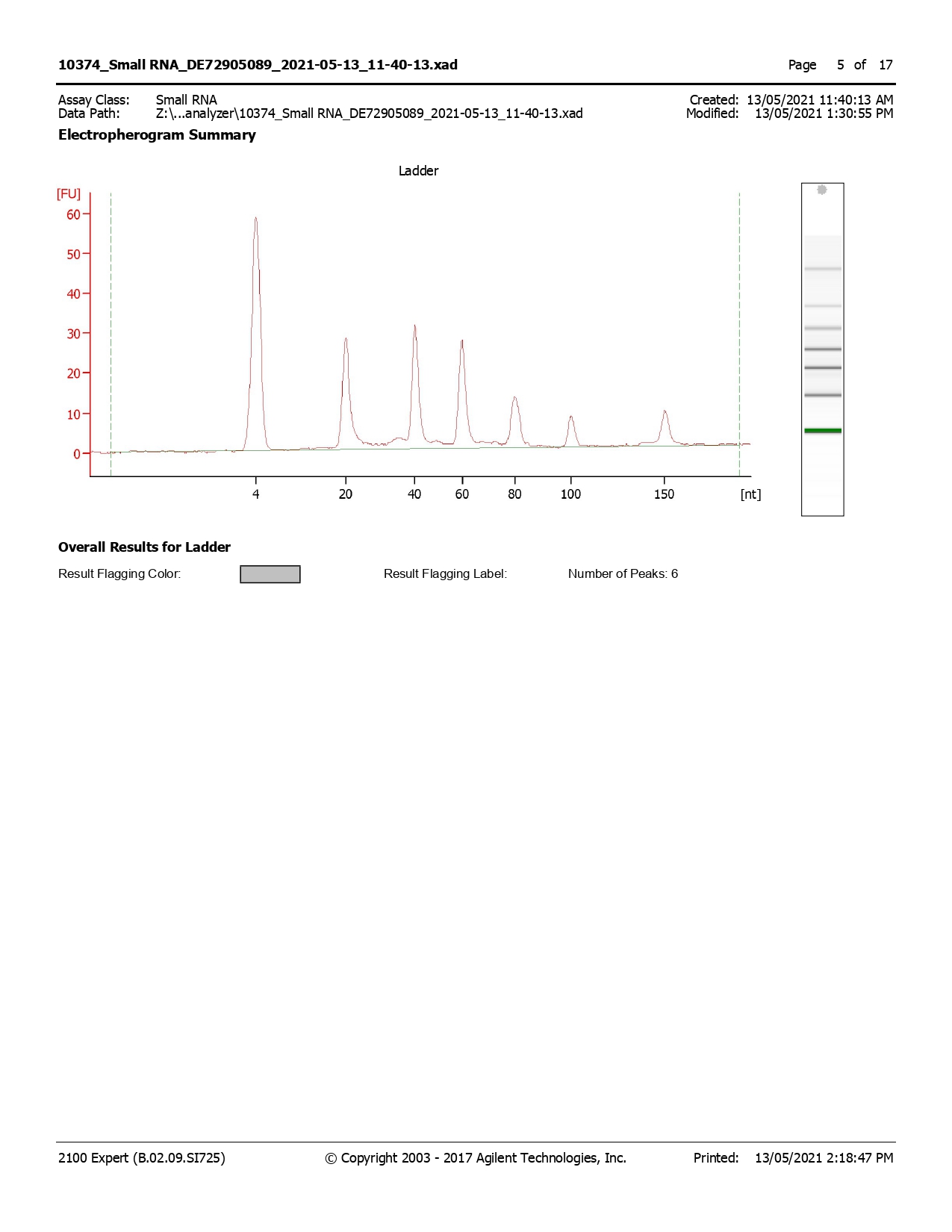

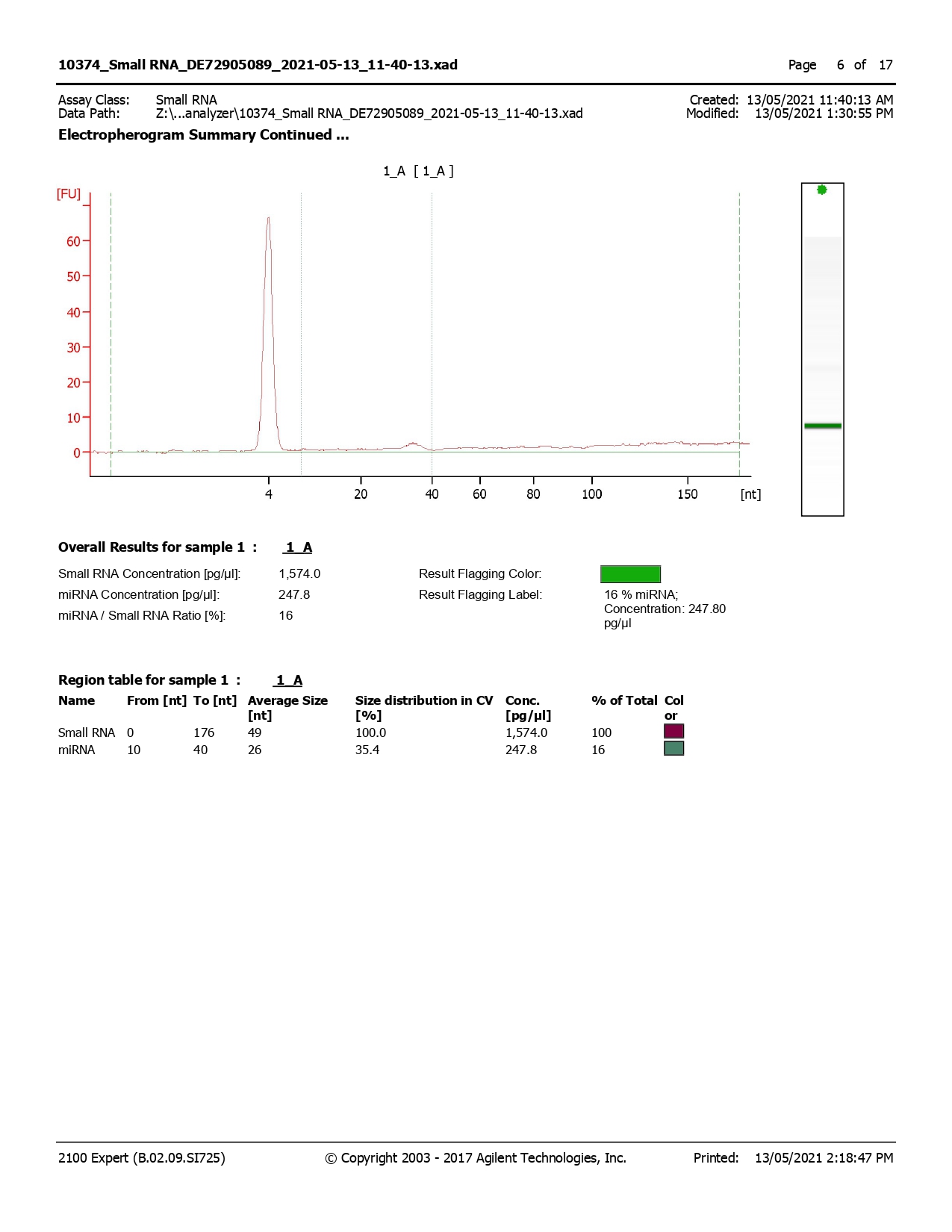

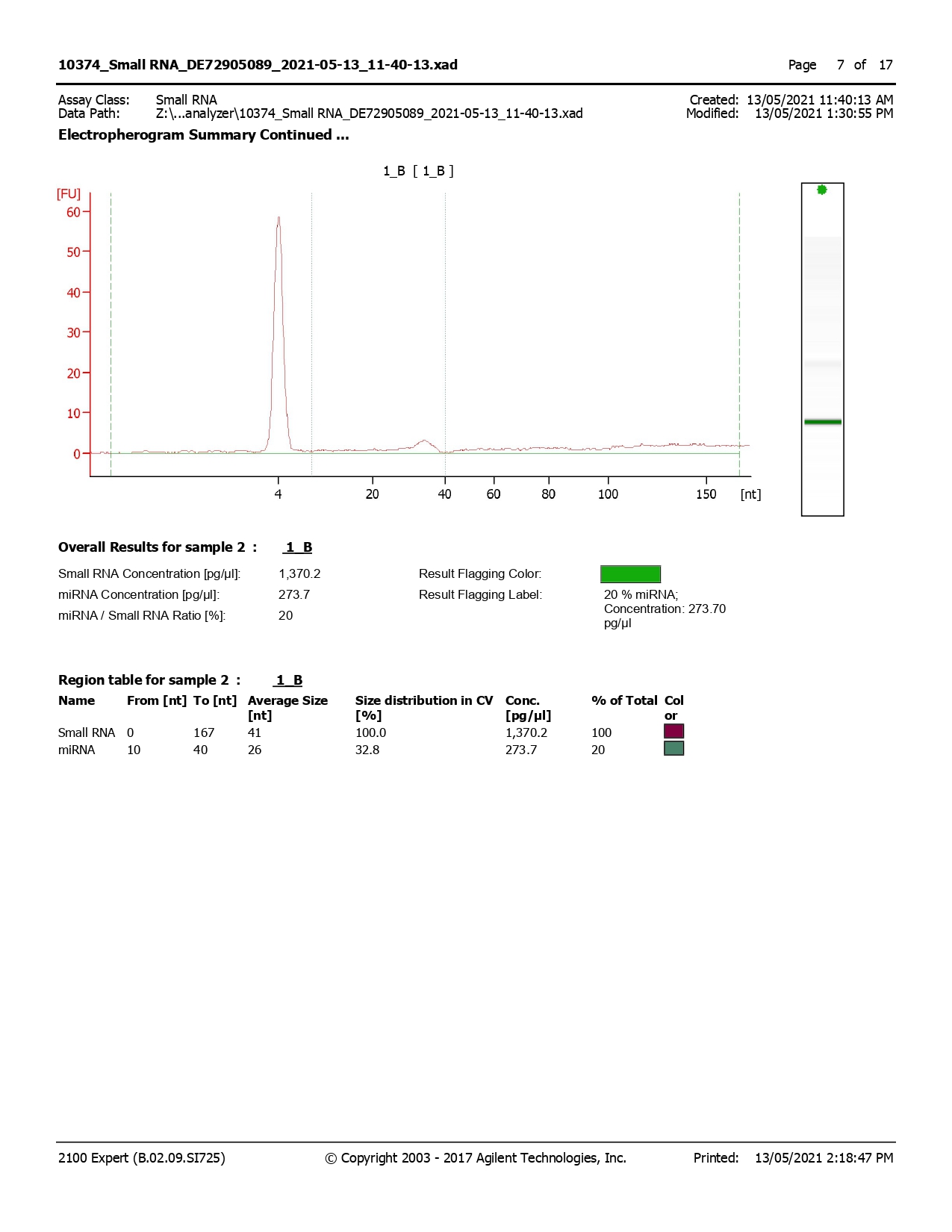

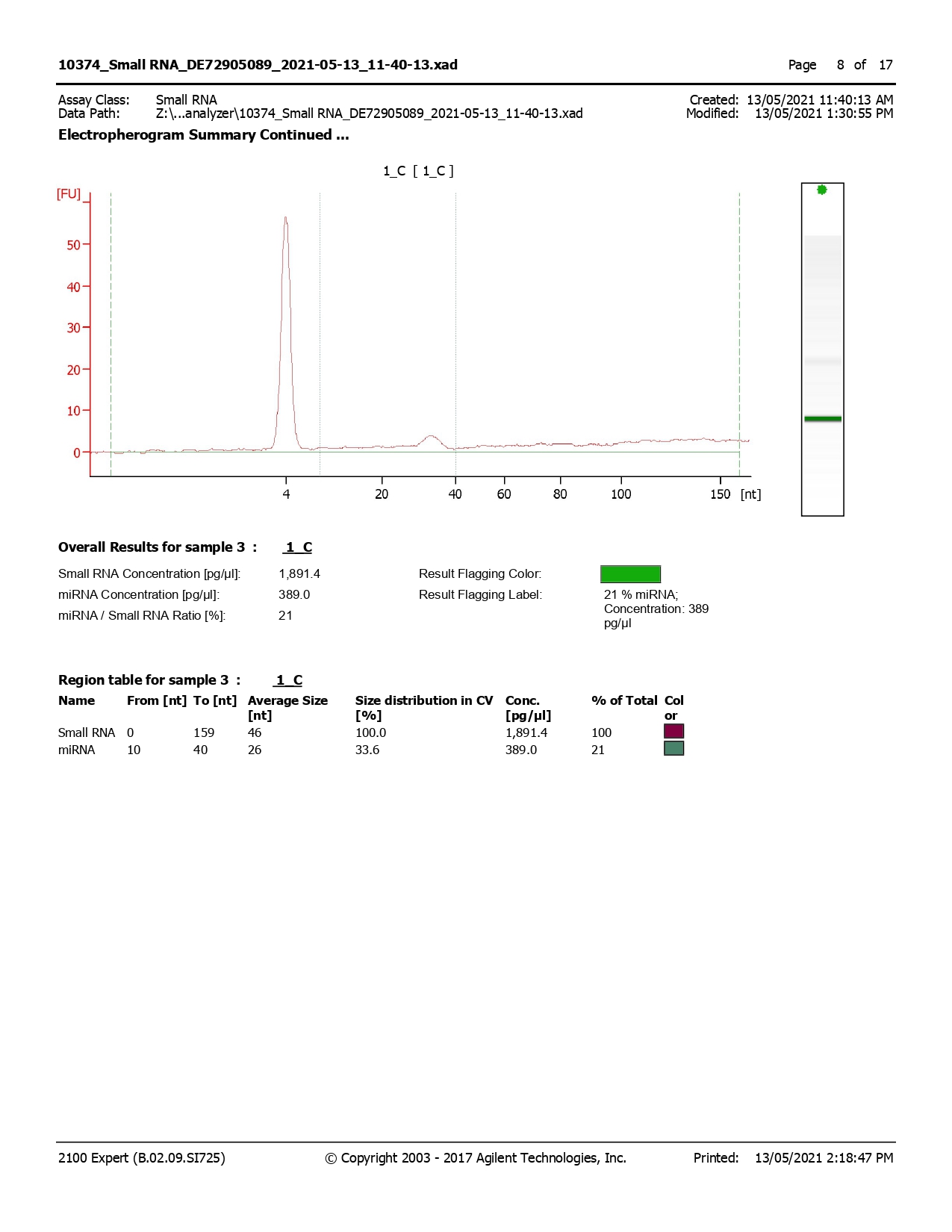

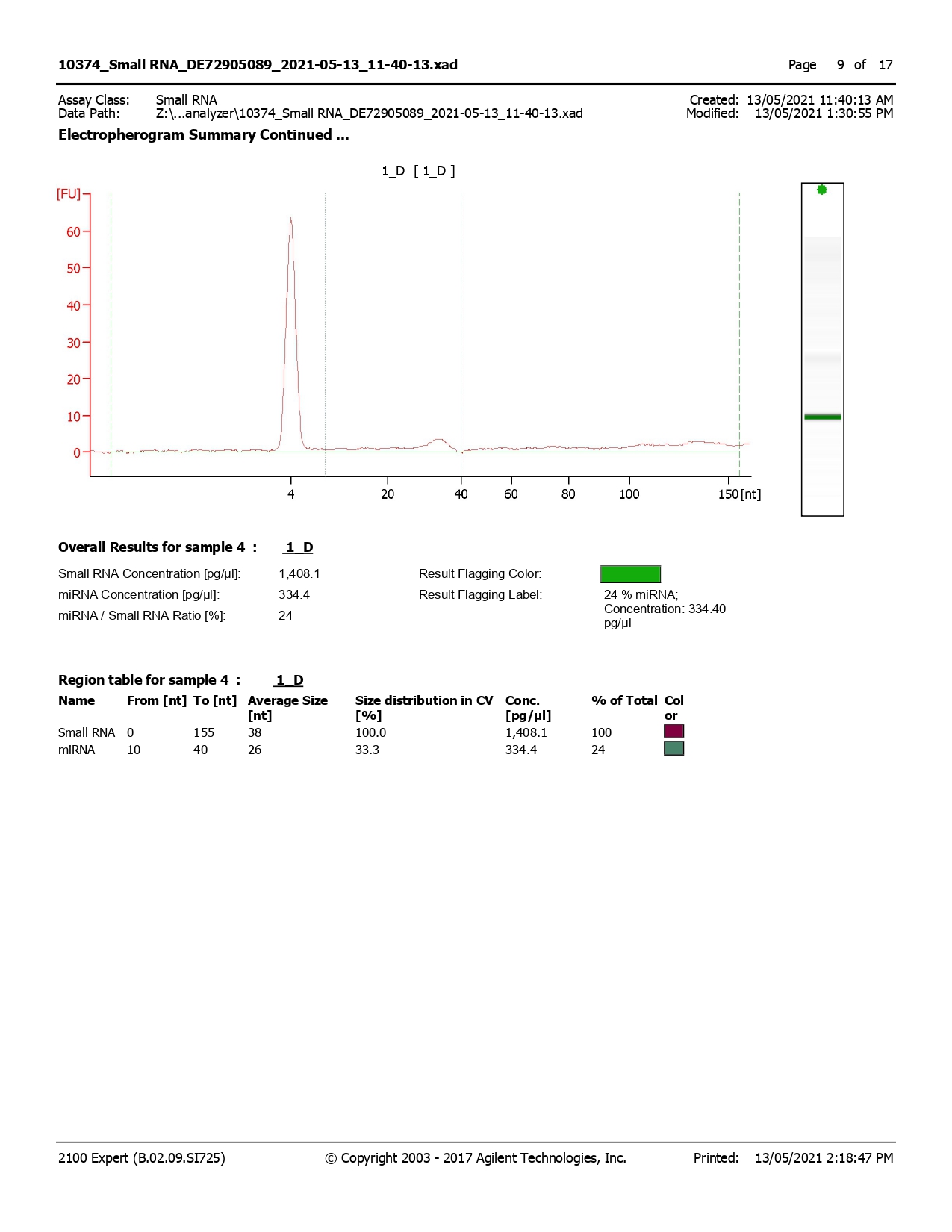

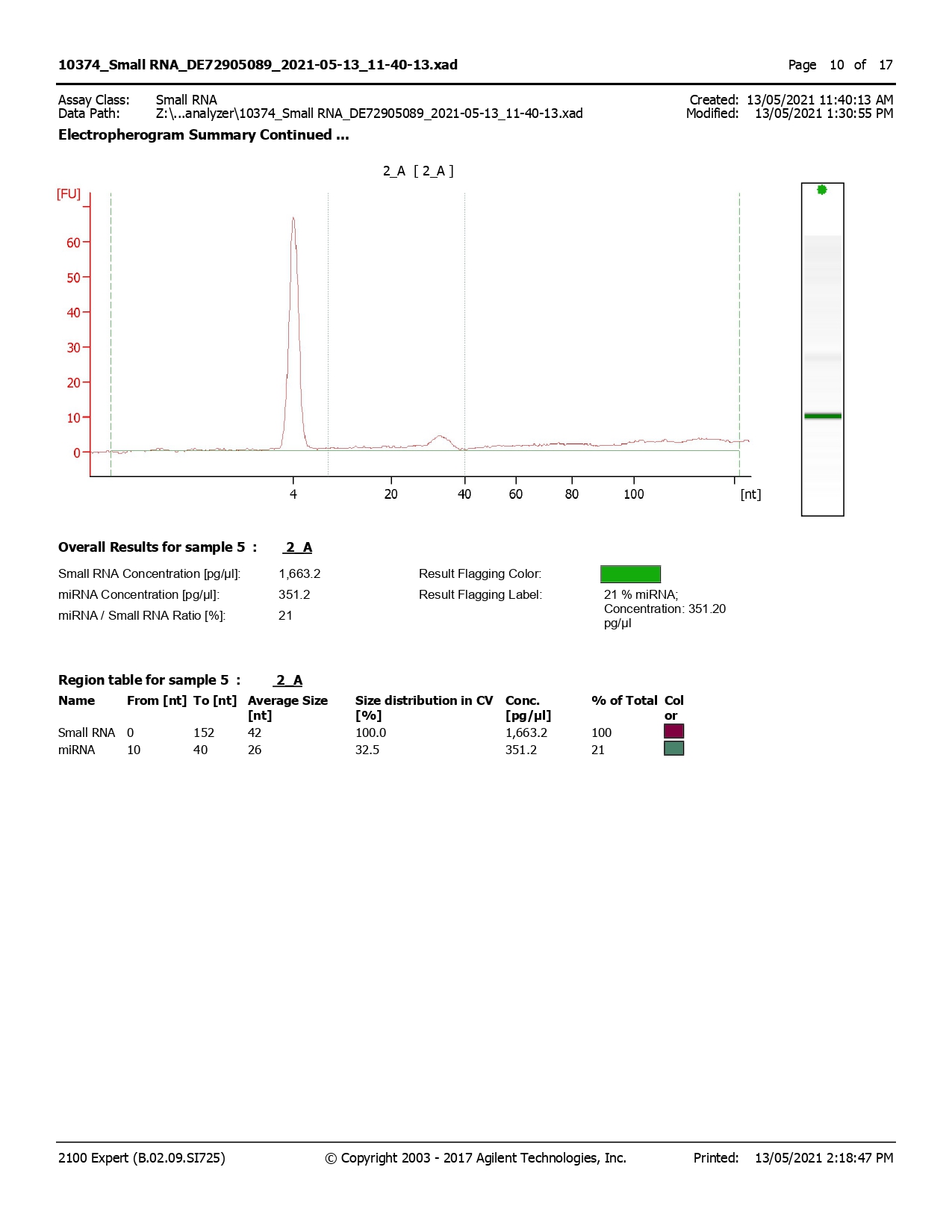

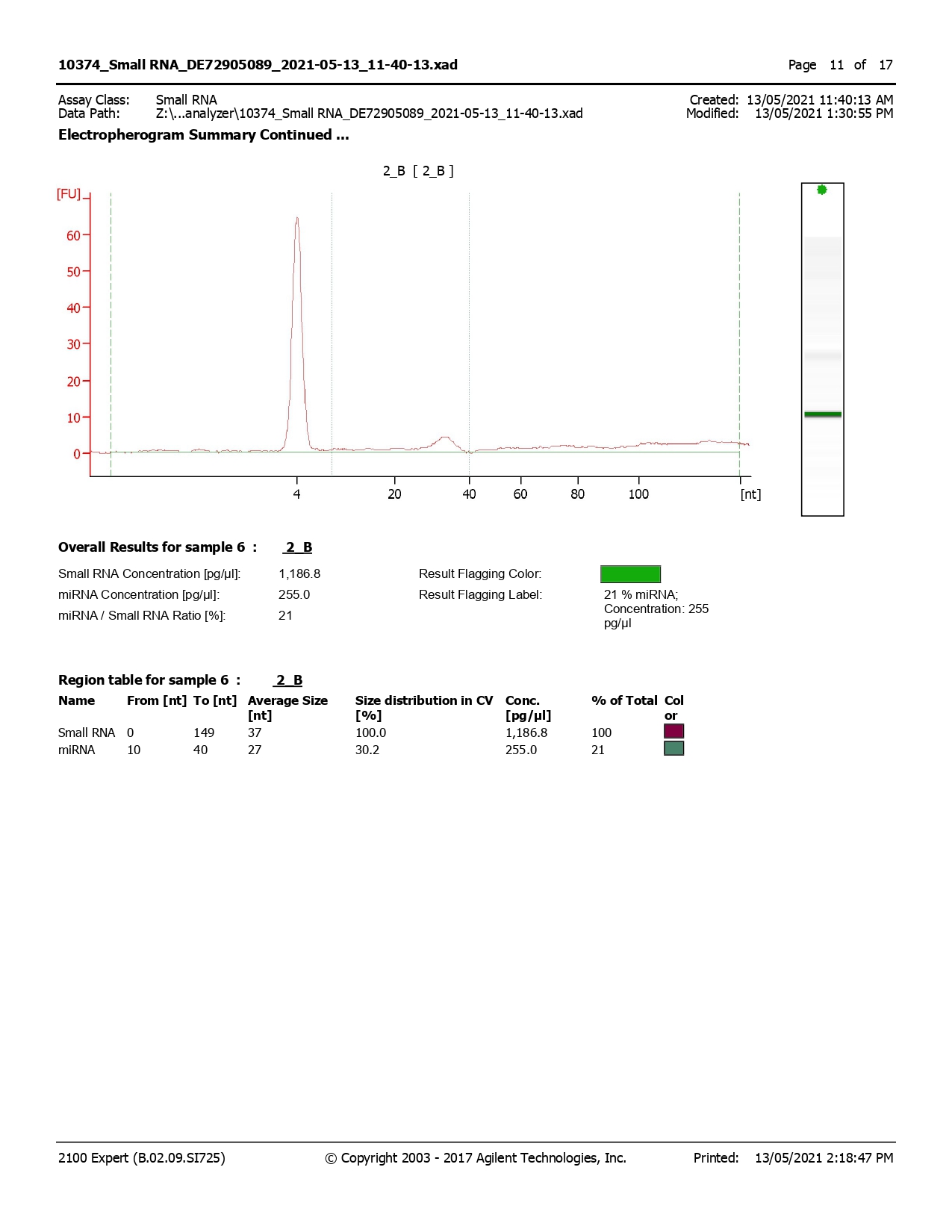

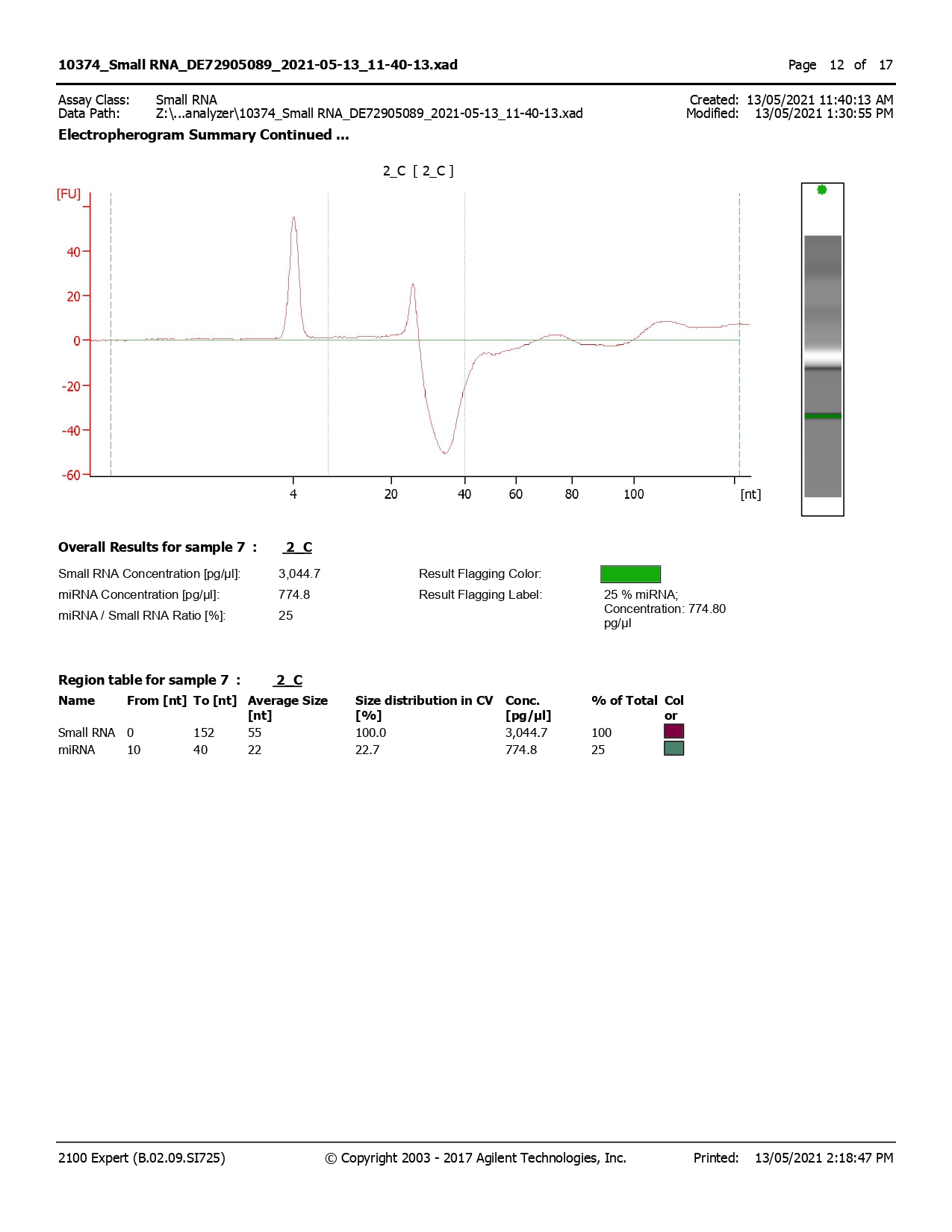

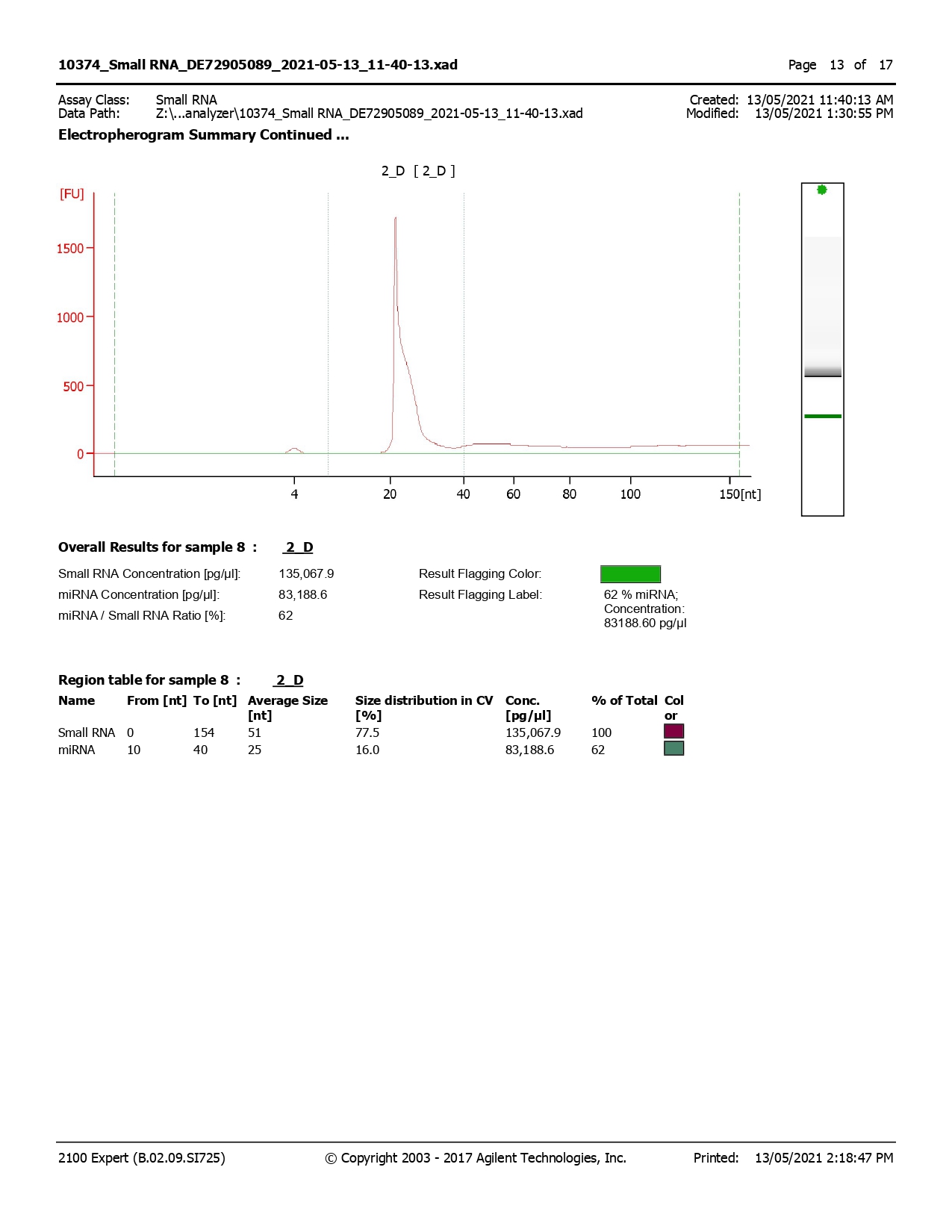

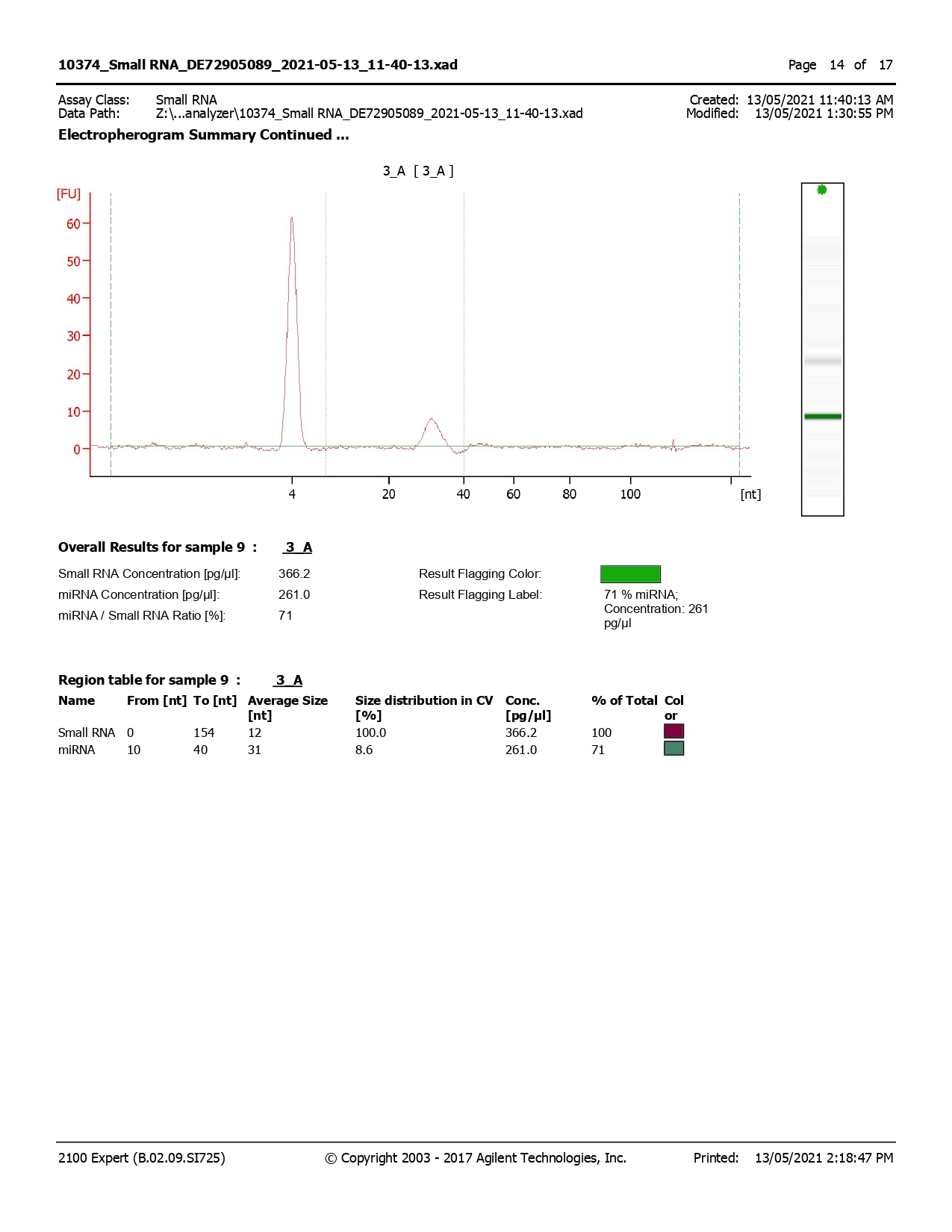

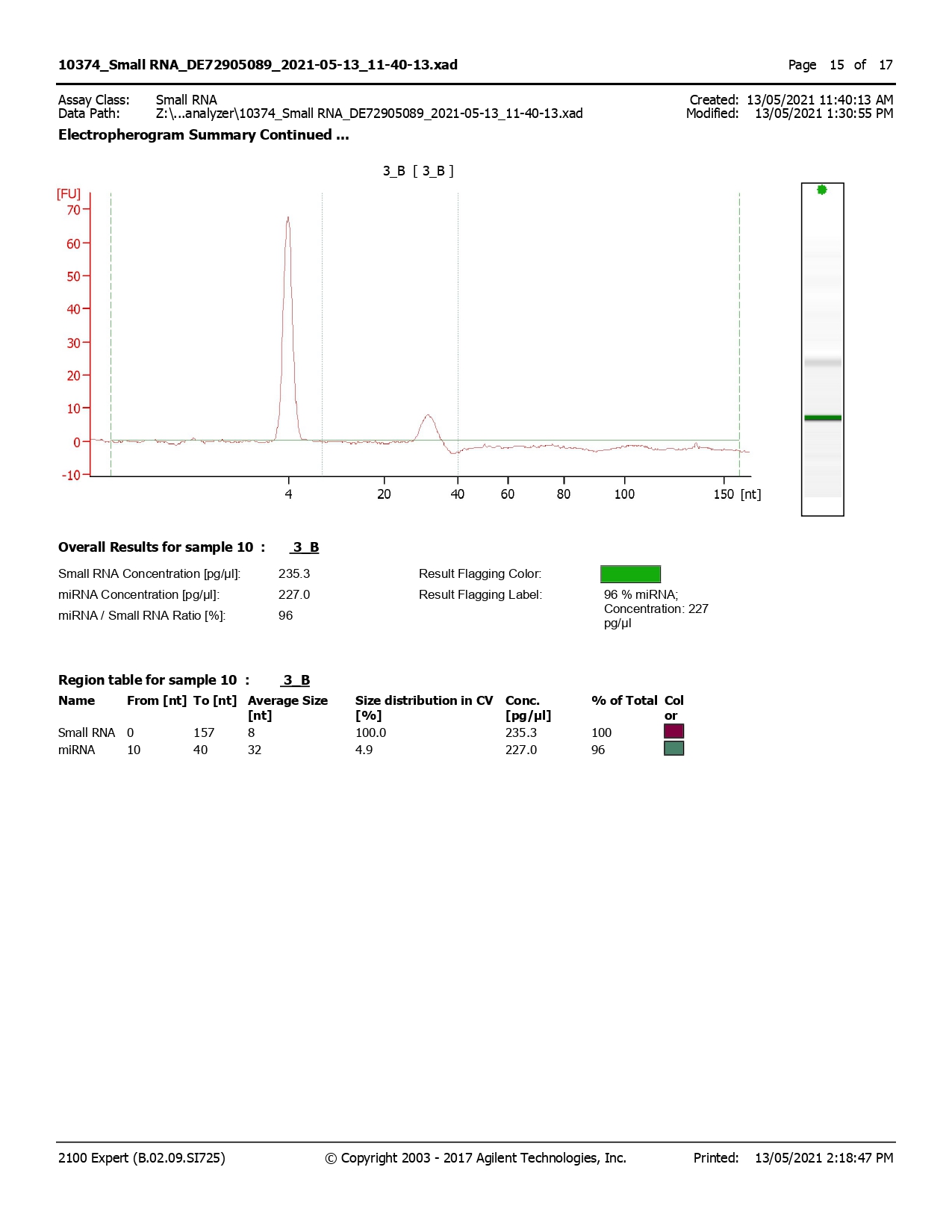

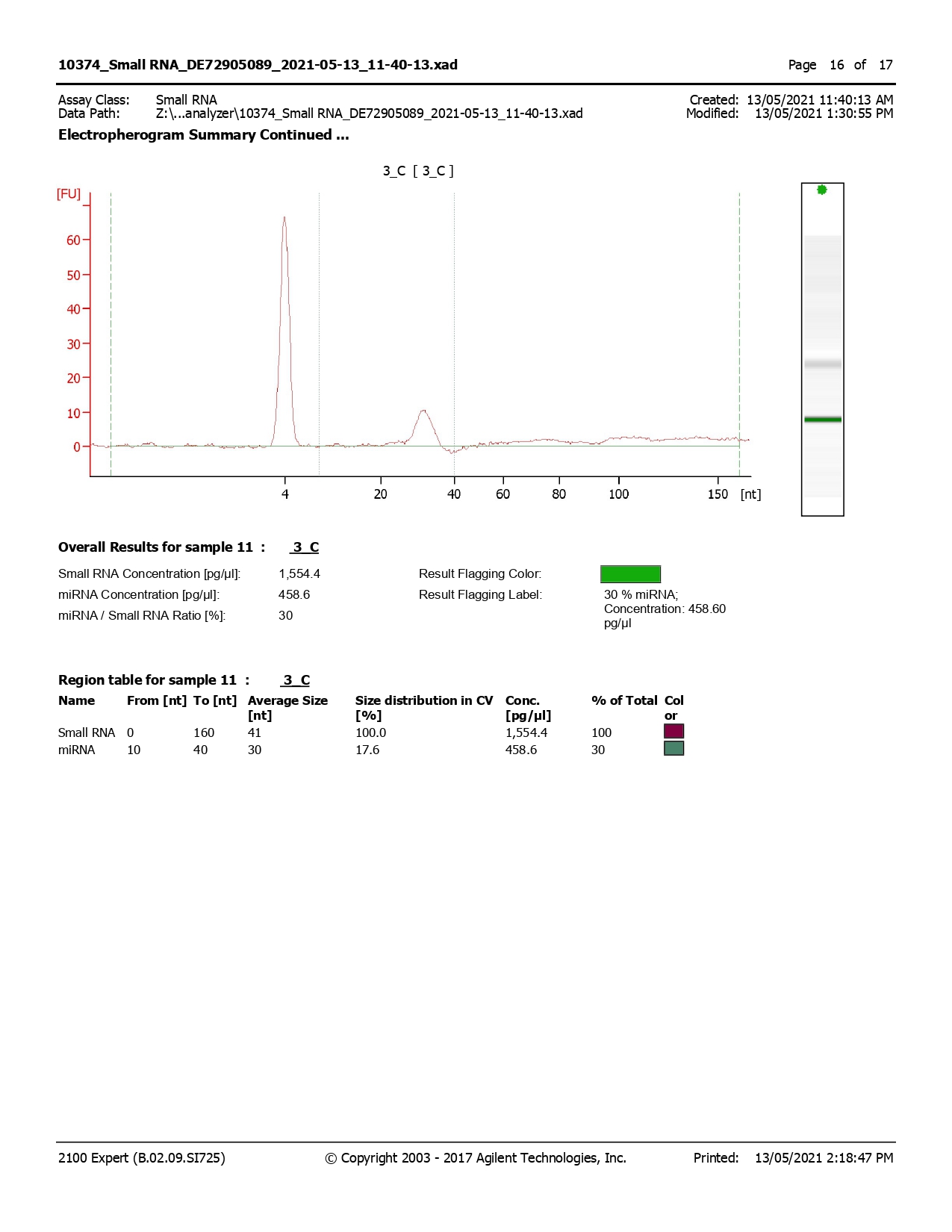


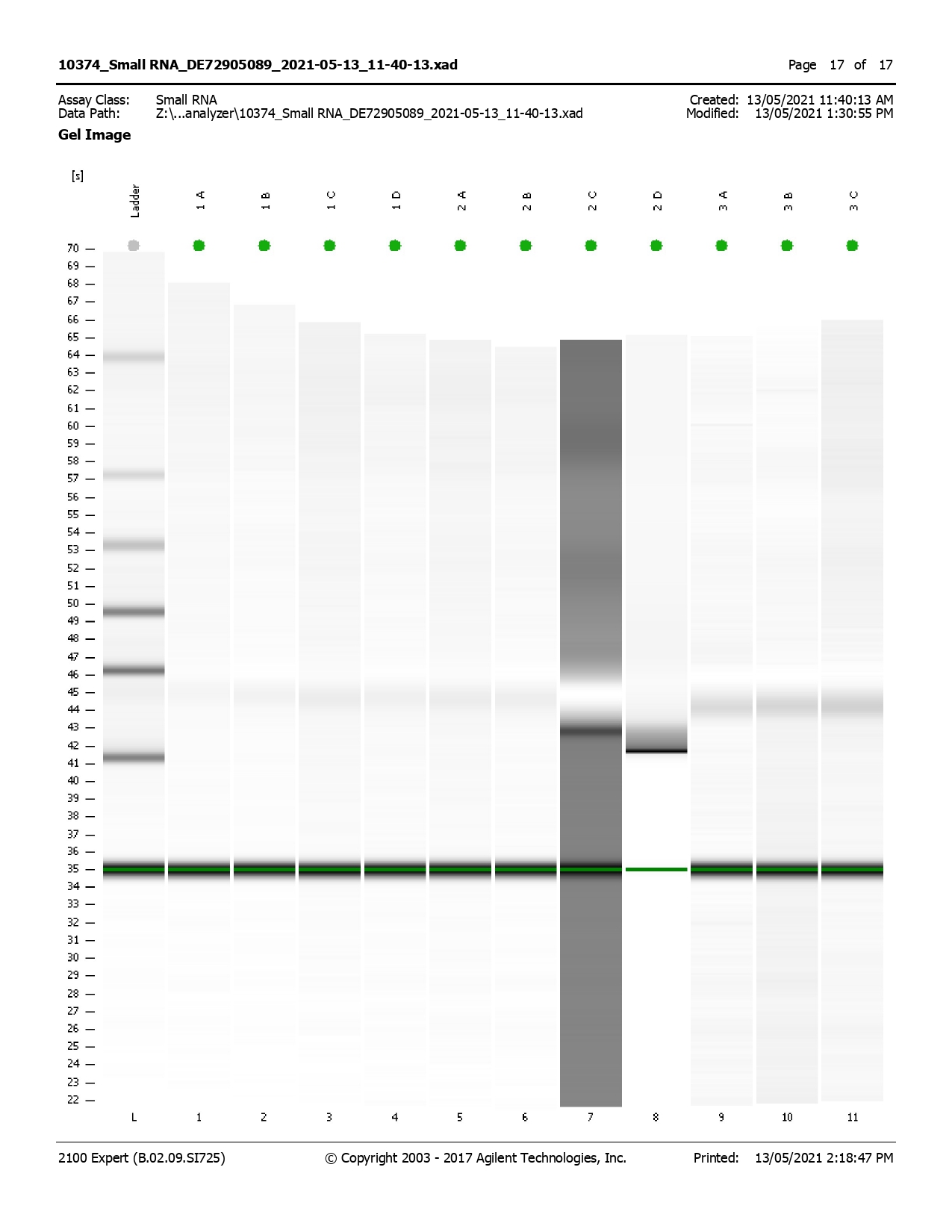


**
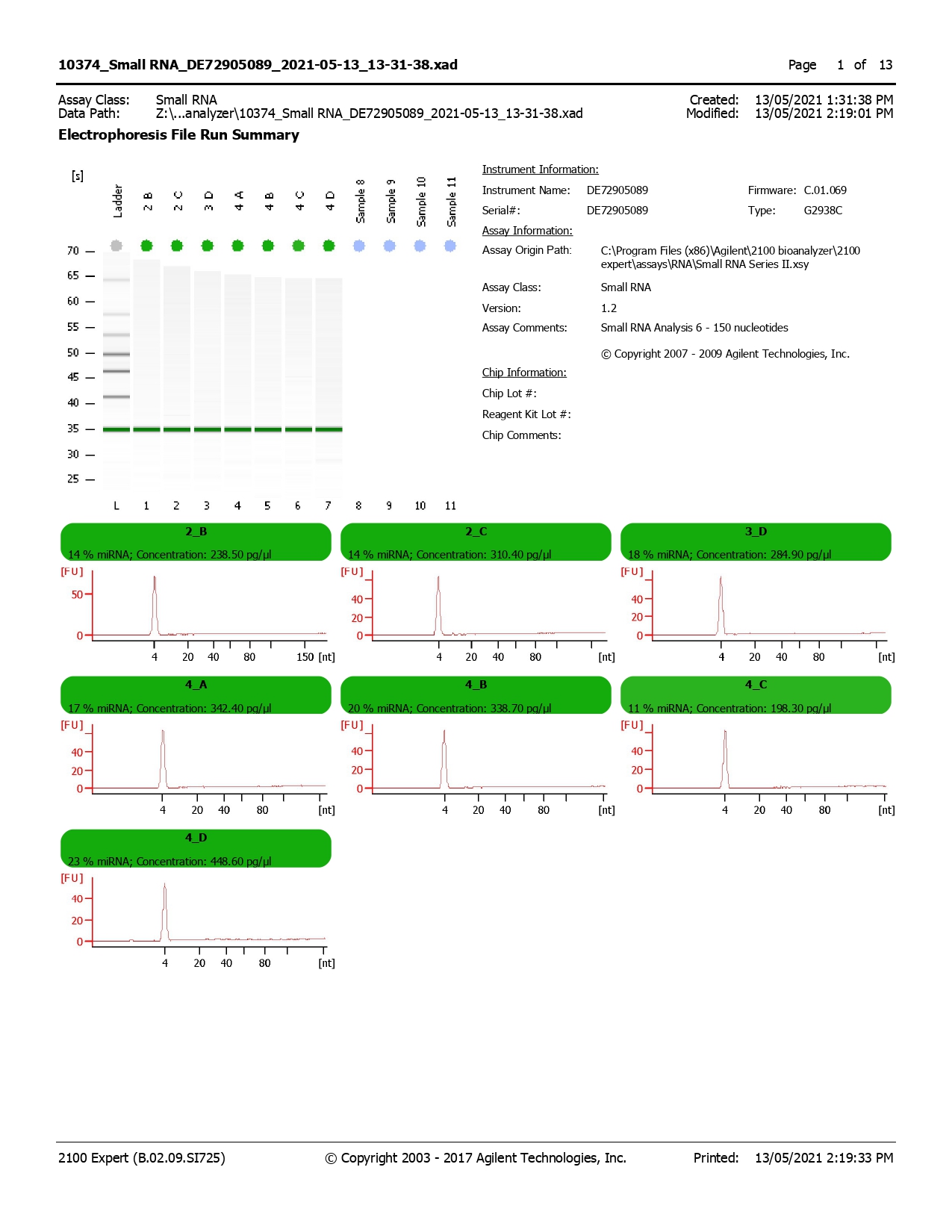
File 2 (page 1-13)**

**
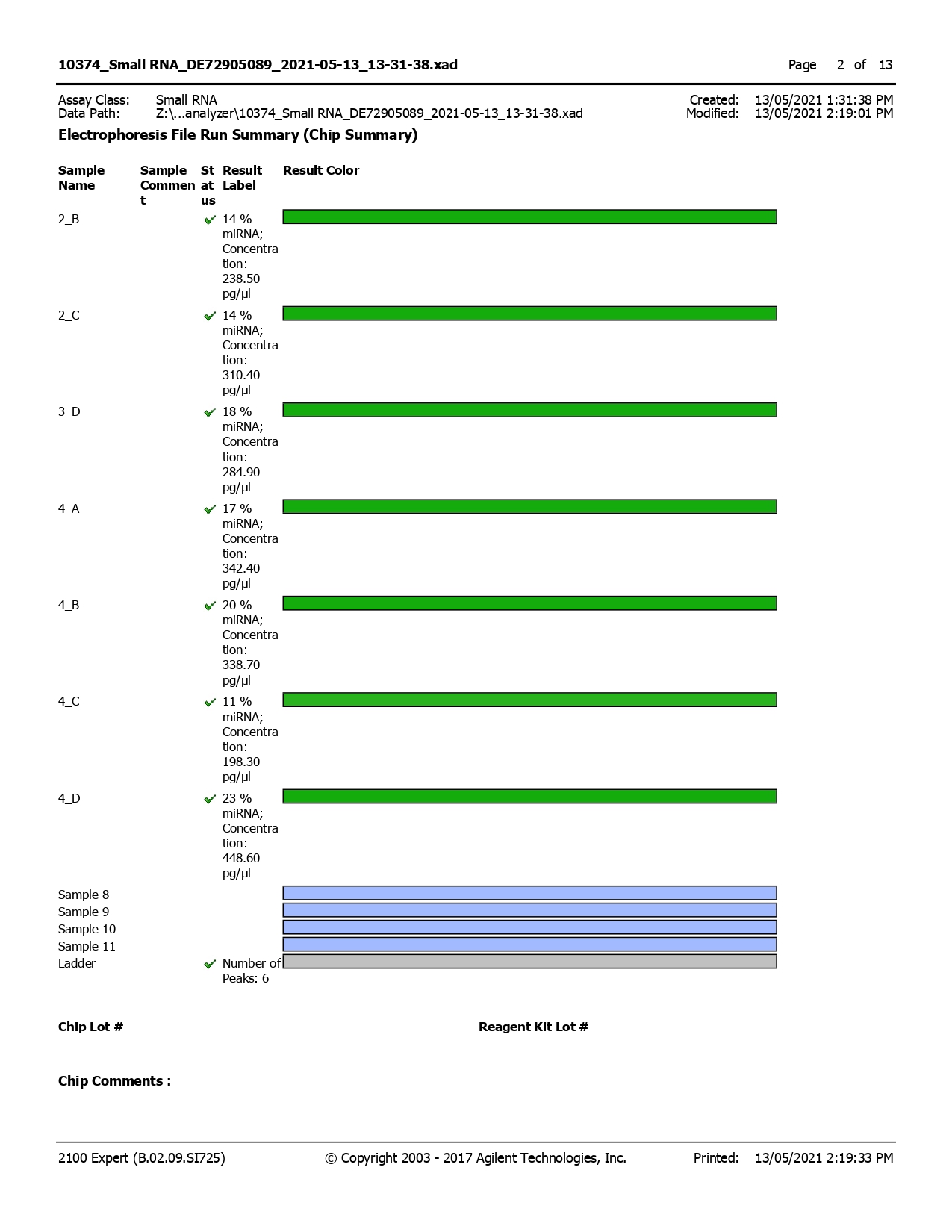

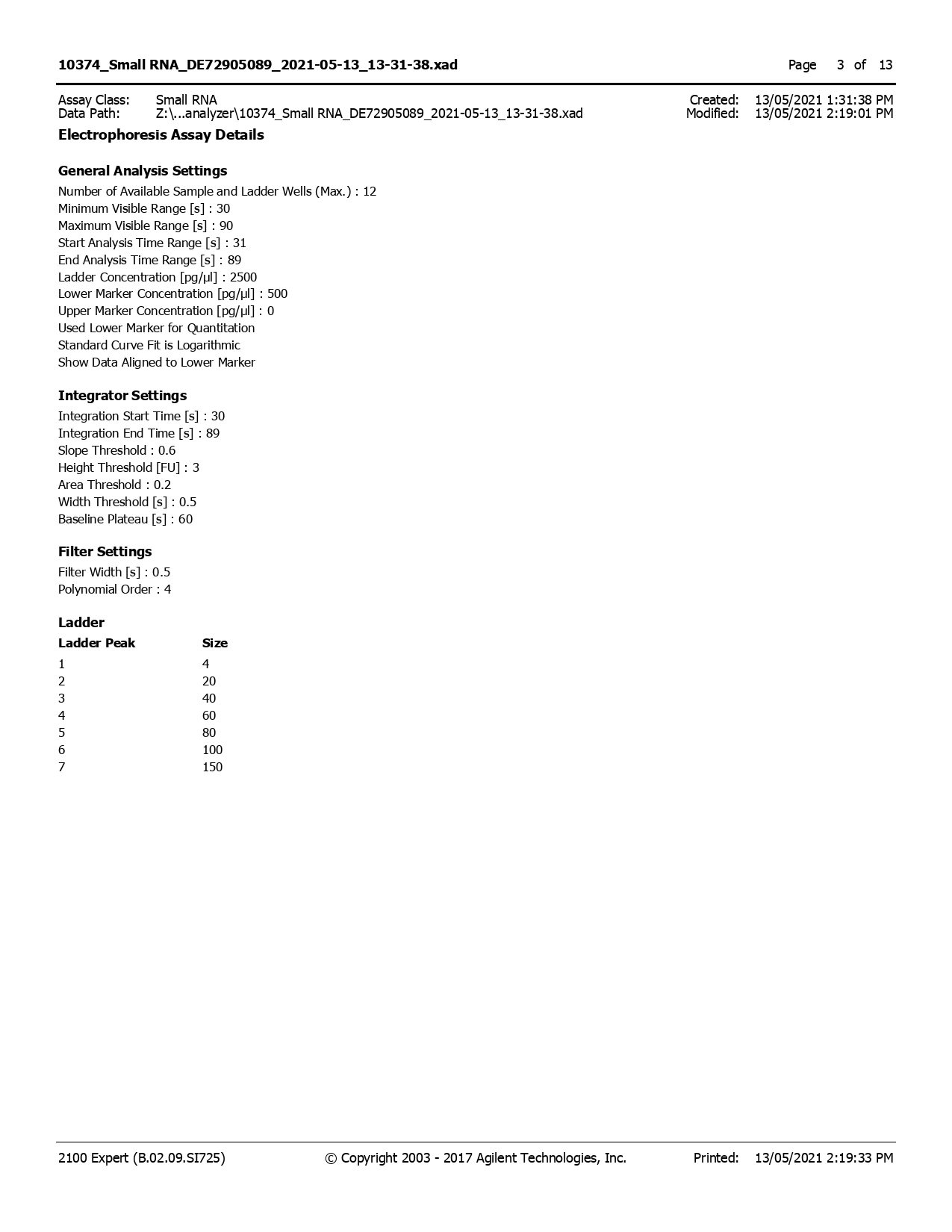

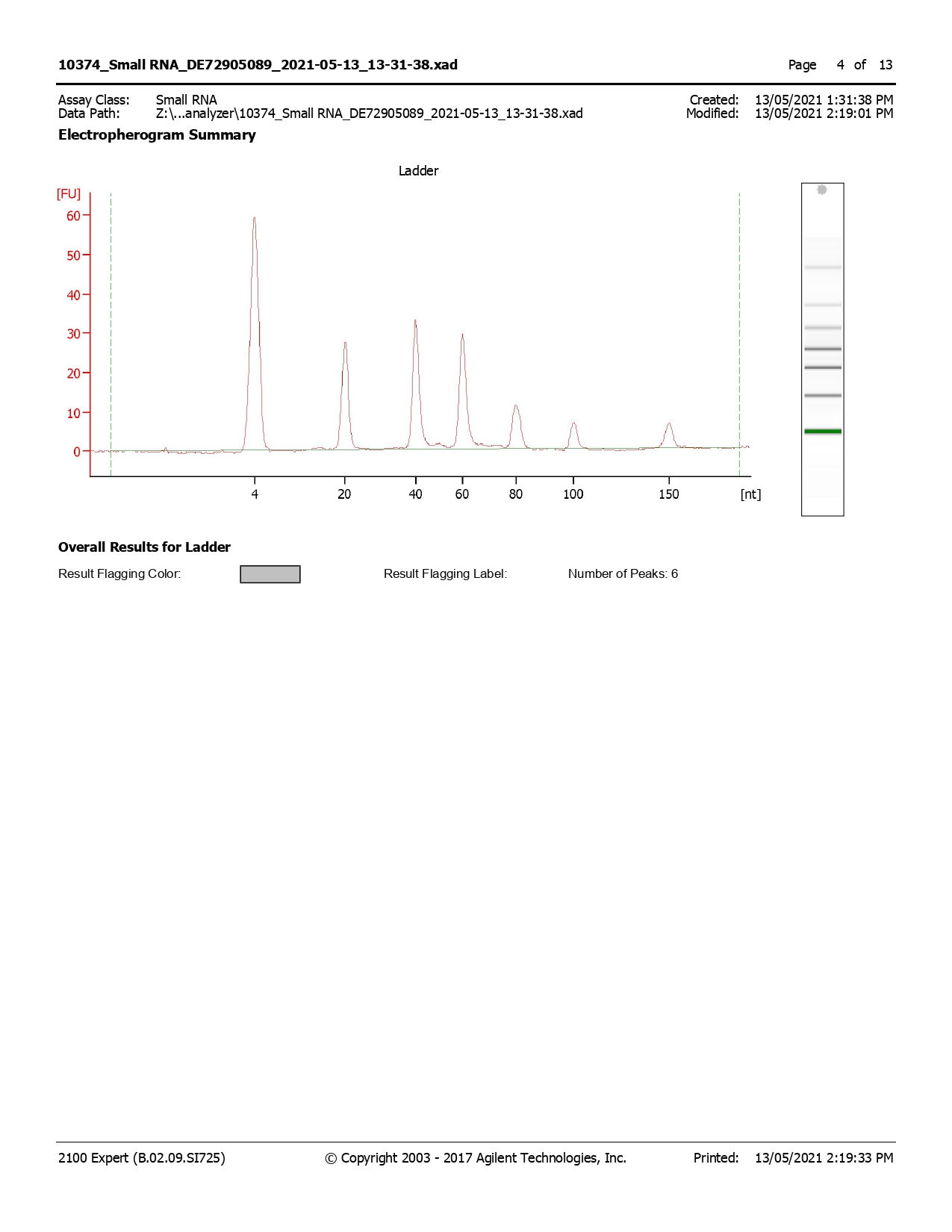

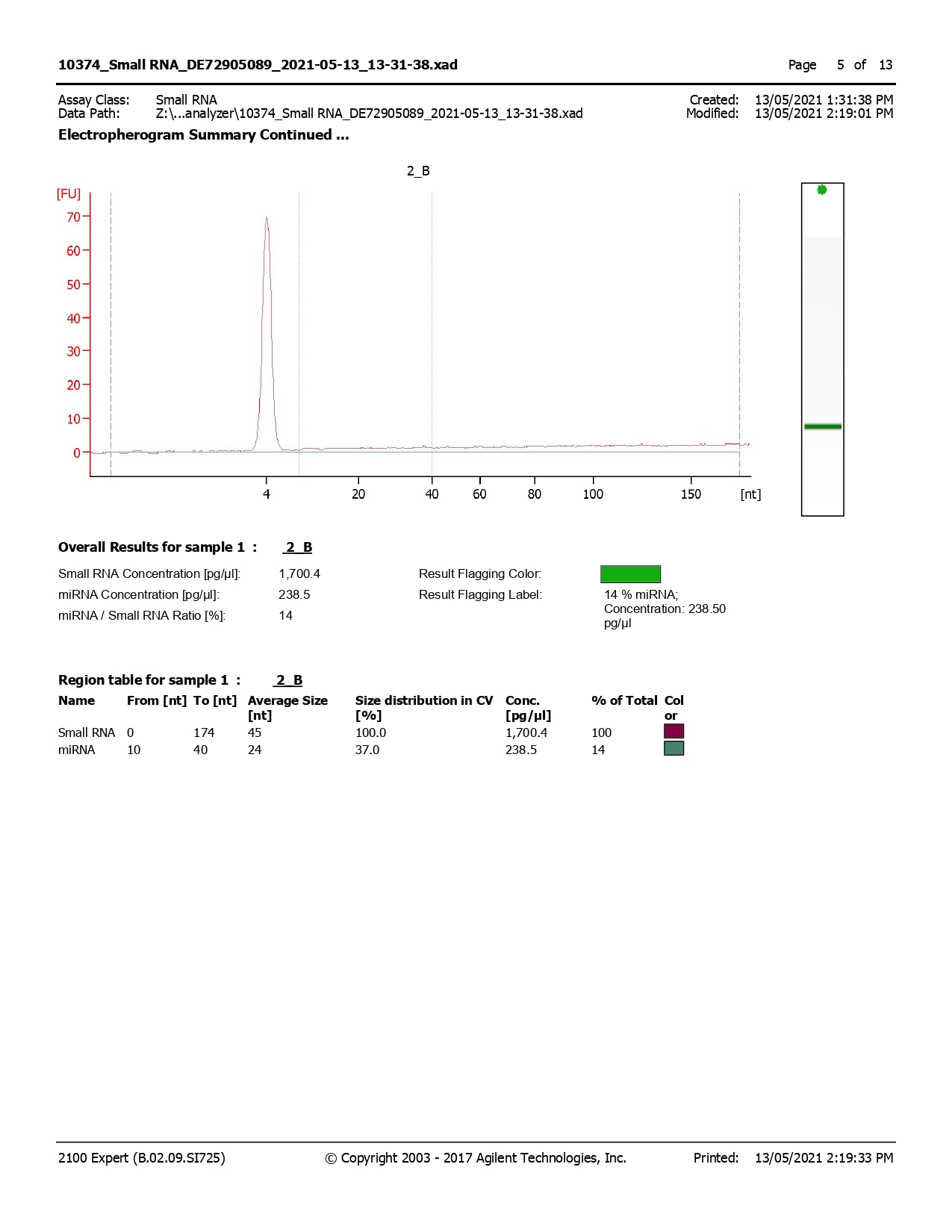

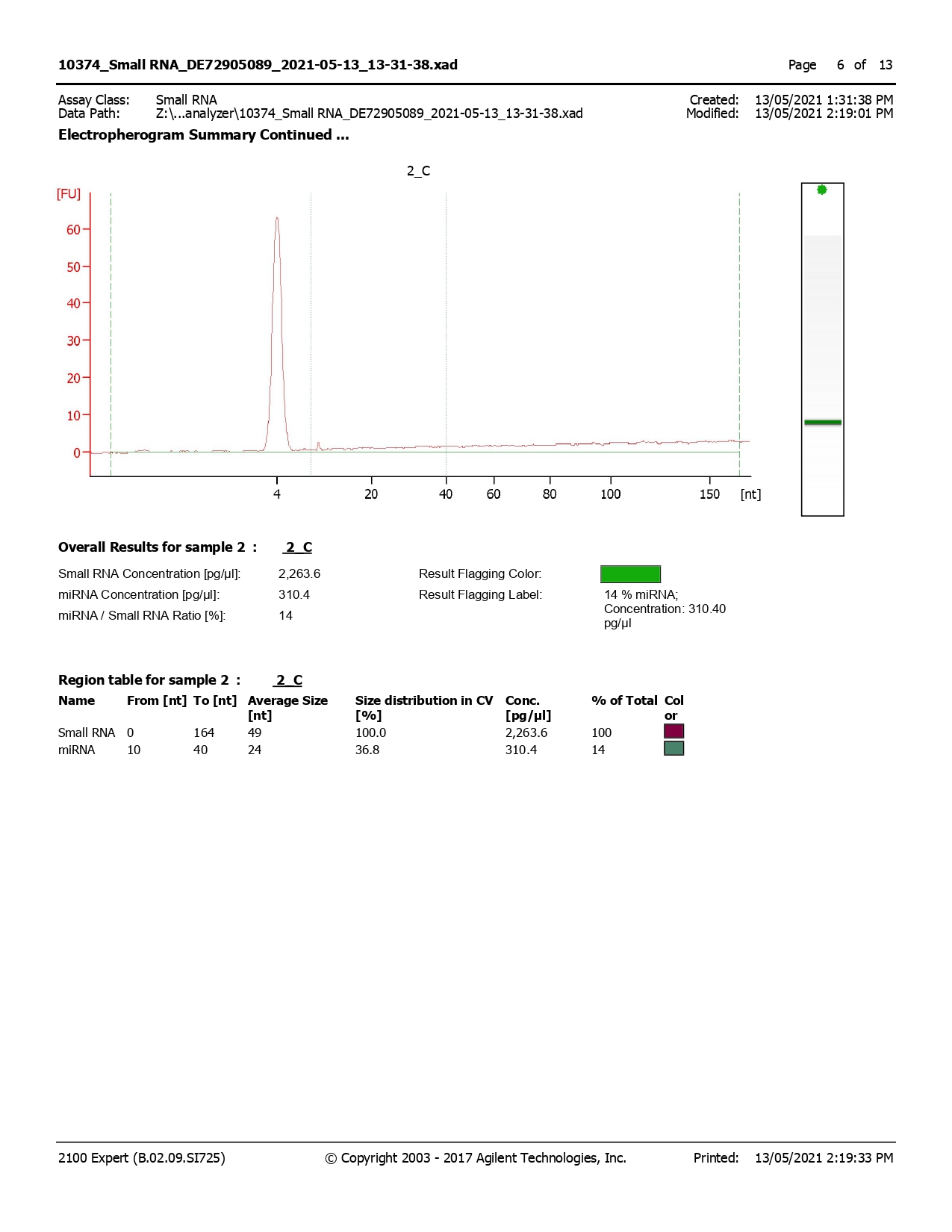

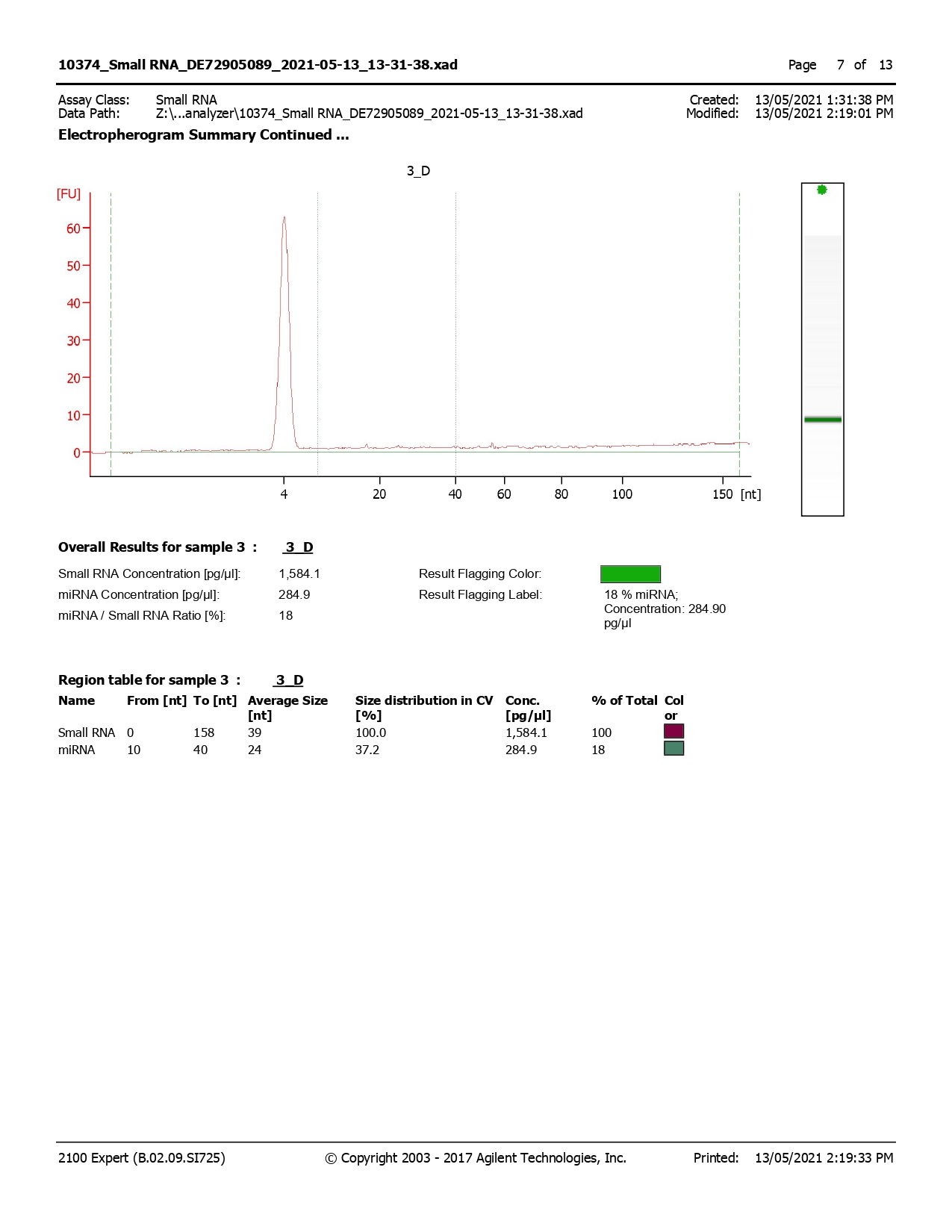

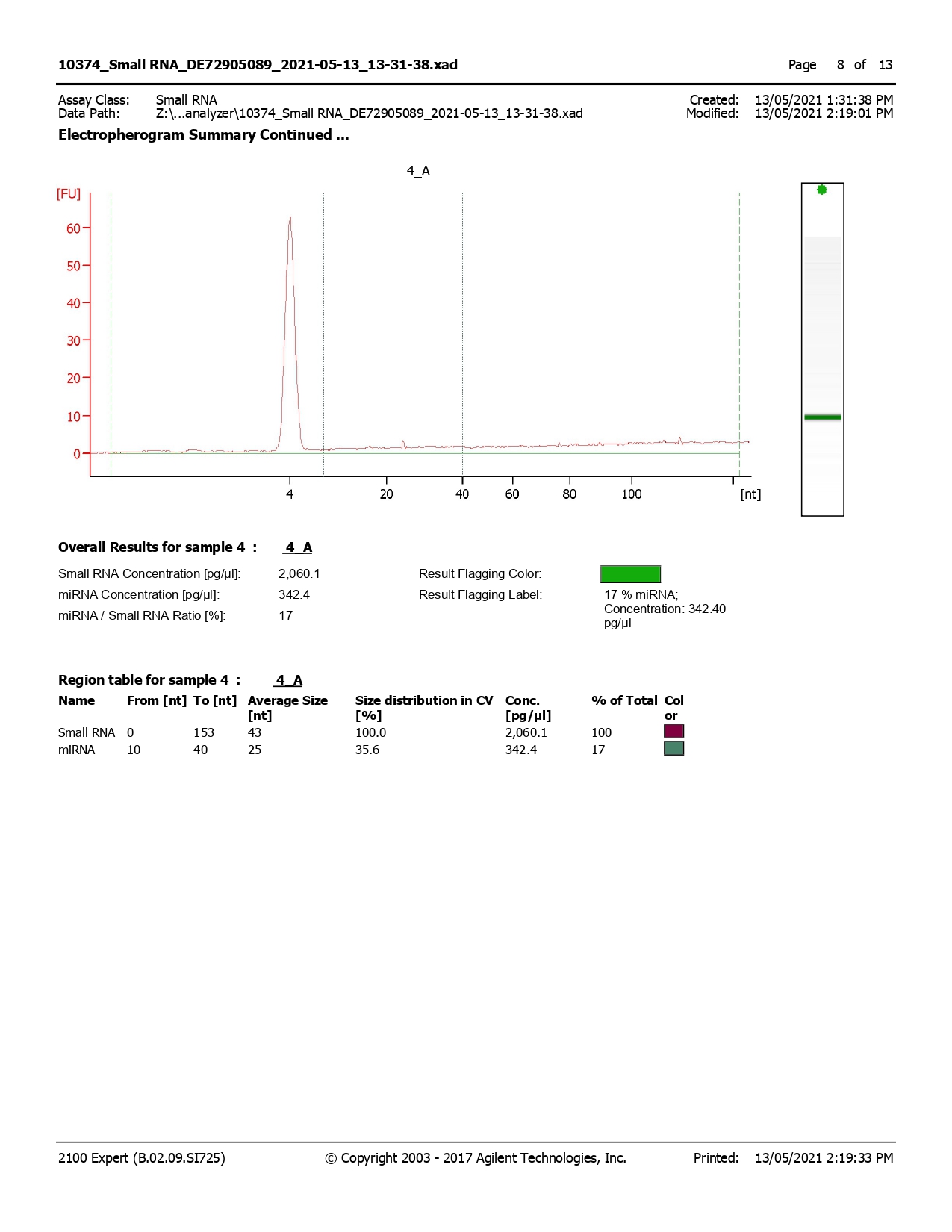

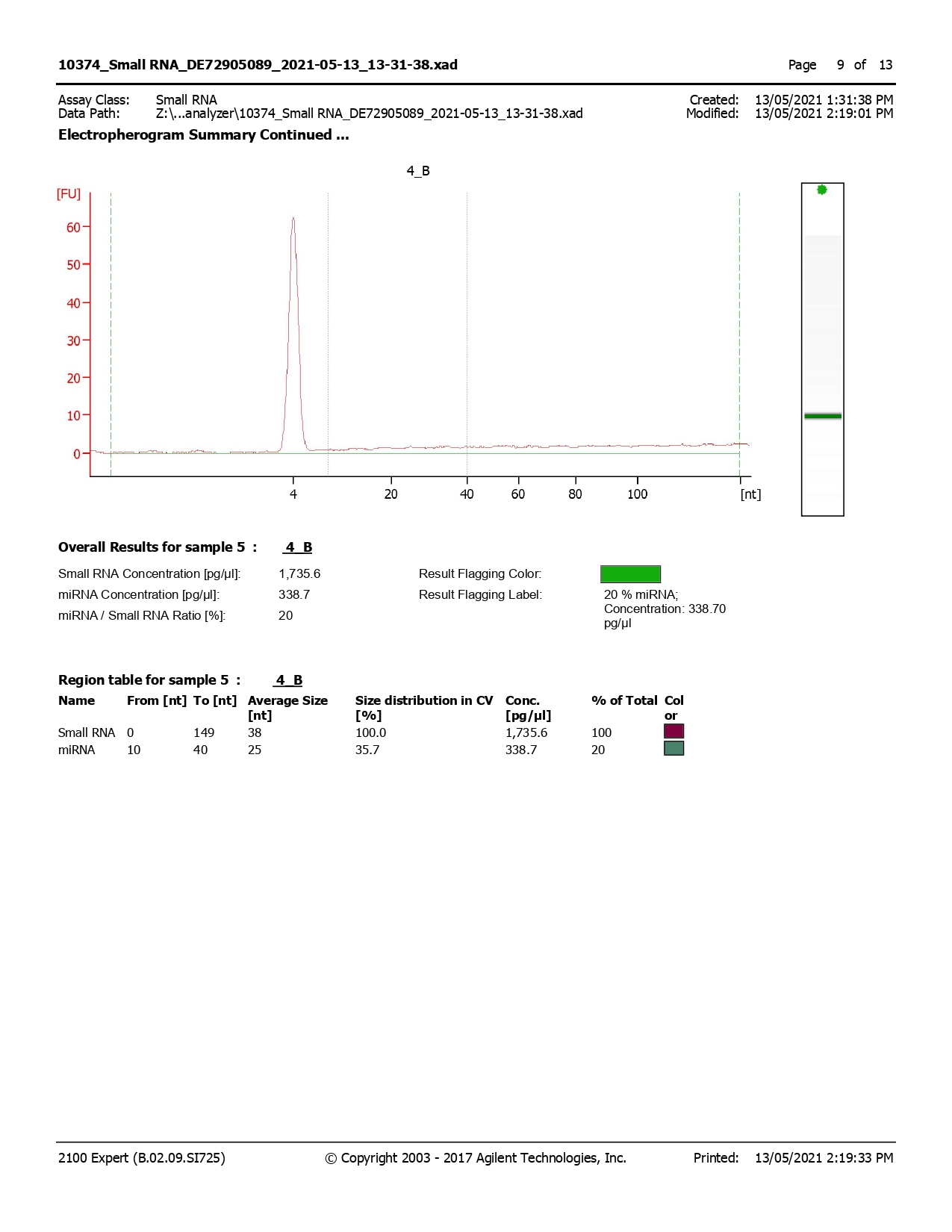

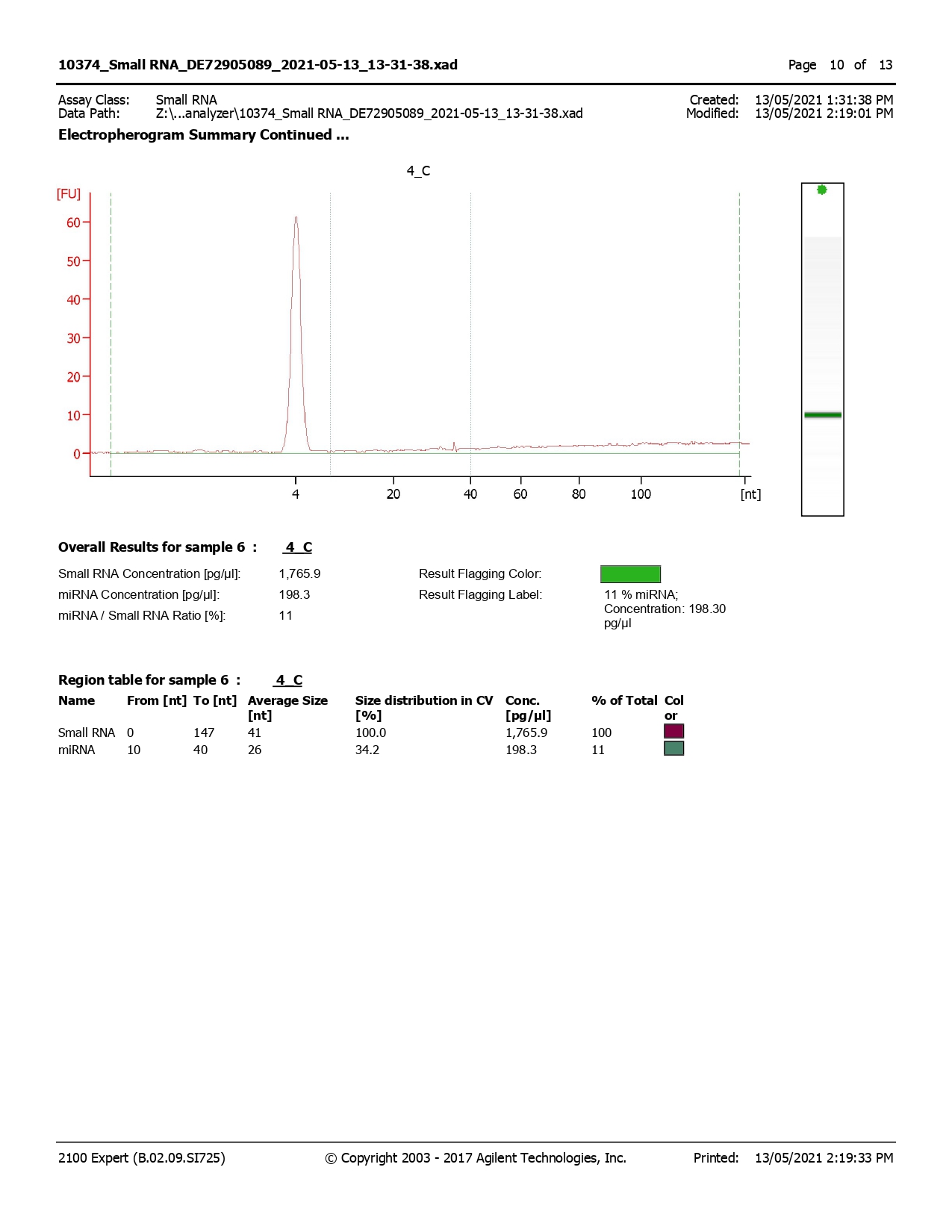

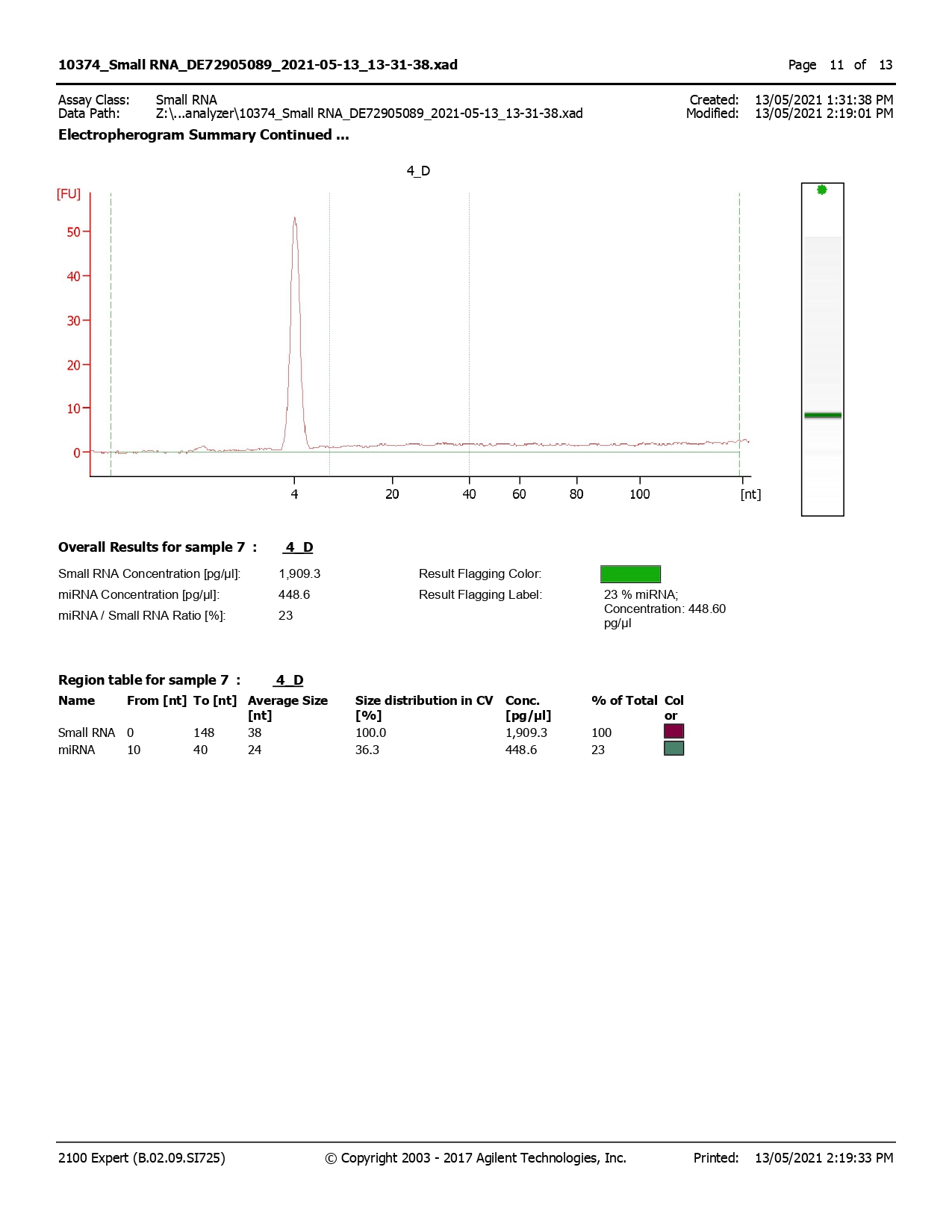

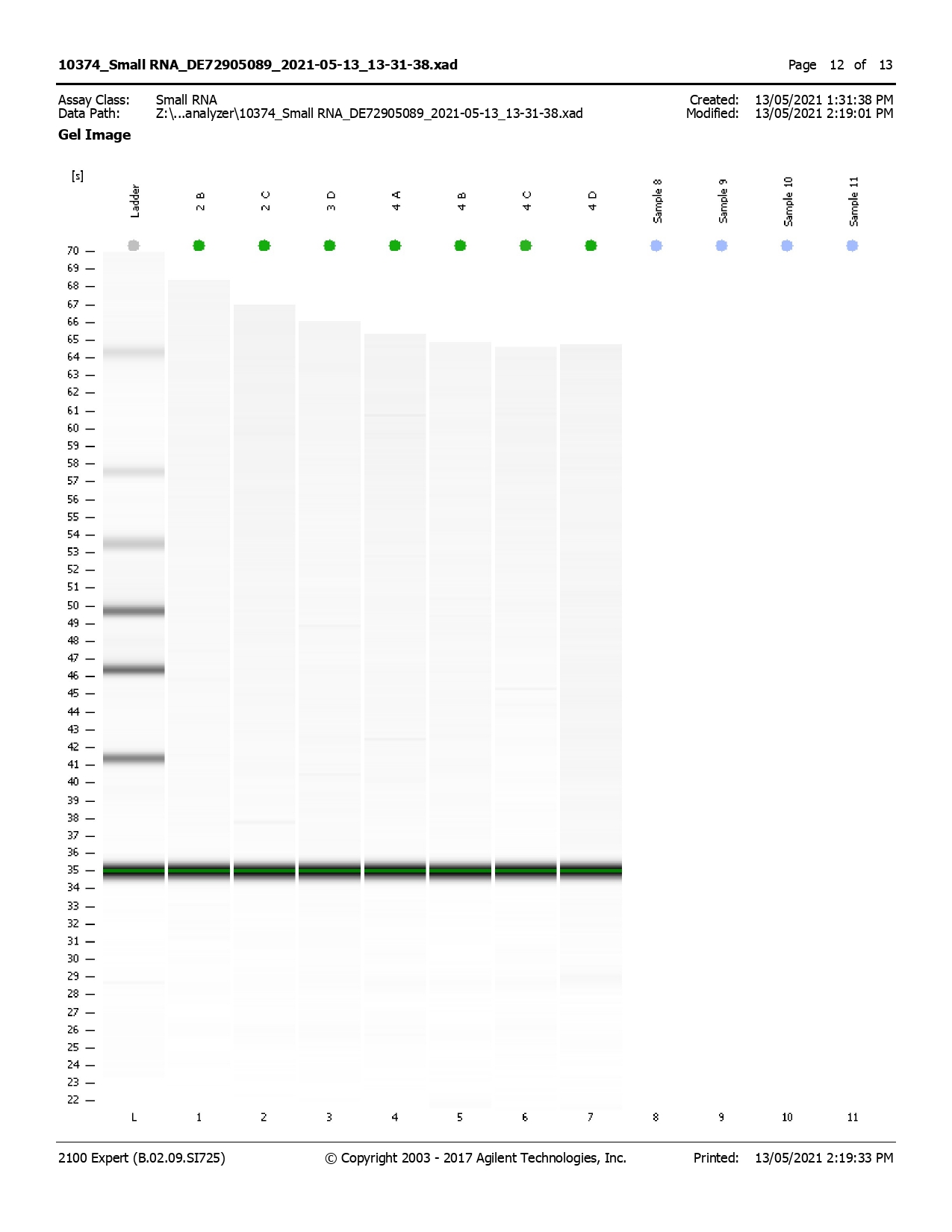

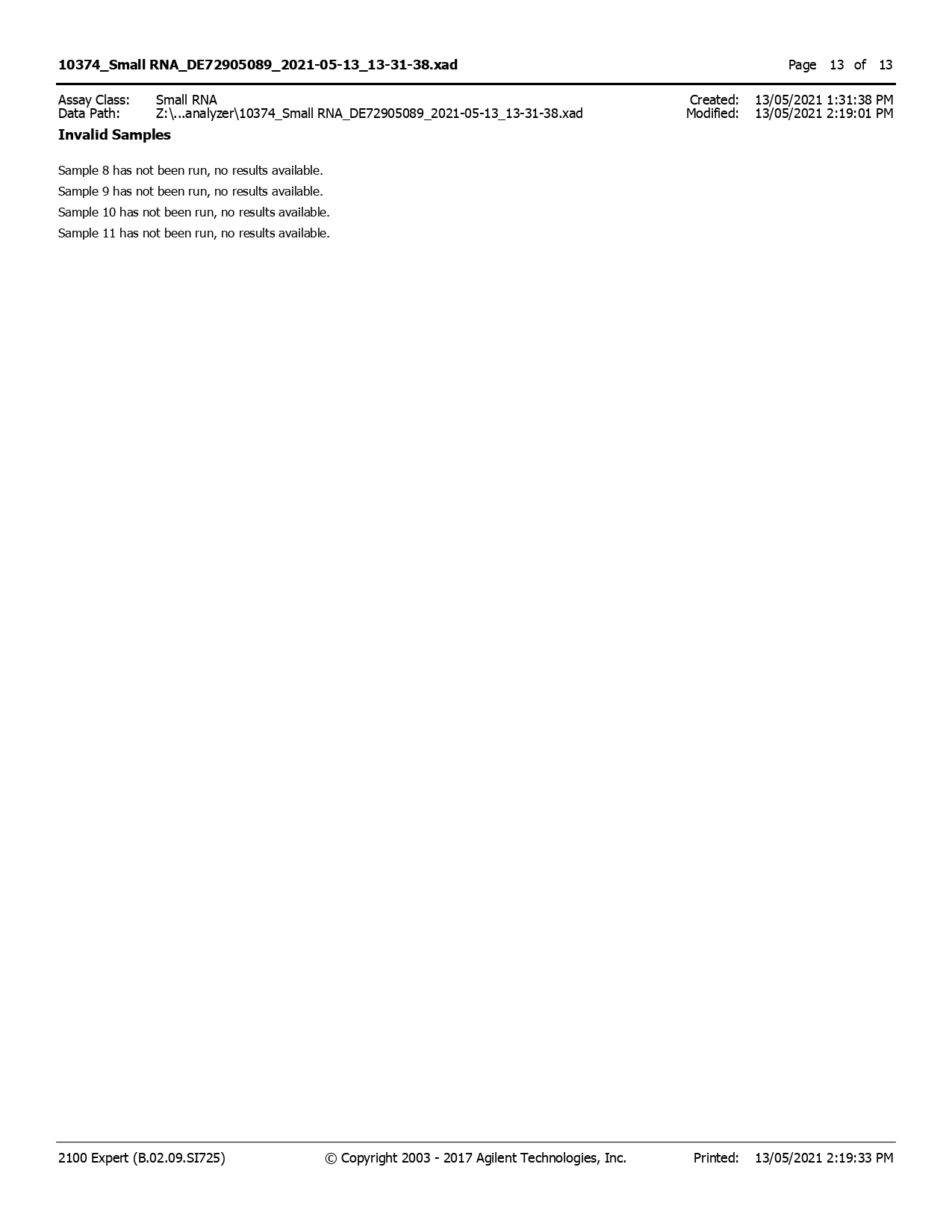
**
