# Supplementary material for: A comparative analysis of small extracellular vesicle (sEV) micro-RNA (miRNA) isolation and sequencing procedures in blood plasma samples
Source: Extracell Vesicles Circ Nucl Acids. 2024 Feb 29;5(1):119–37. doi: 10.20517/evcna.2023.55 (PMC11648519; doi:10.20517/evcna.2023.55)
Supplement: Supplementary file 1 [file evcna-5-1-119-SupplementaryMaterials.zip › evcna5055-SupplementaryMaterials/Supplementary File 2.docx]

Bioinformatics pathway

setwd("C:/R")

library(edgeR)

library( gplots )

library(RColorBrewer)

matrix <- read.csv("count_table_methods.csv", header=TRUE)

head(matrix)

matrix_filter <- matrix[,-(1)]

head(matrix_filter)

rownames(matrix_filter) <- matrix[,1]

head(matrix_filter)

colnames(matrix_filter)

list <- matrix_filter

colnames(list)

y <- DGEList(list)

head (y)

names (y)

y$samples

group <- factor( c("BPControl","UC_SEC","UC_SEC","UC_SEC","UC_SEC","SEC_UC","SEC_UC","SEC_UC","SEC_UC","SEC_UF","SEC_UF","SEC_UF","SEC_UF","UC_SEC_UF","UC_SEC_UF","UC_SEC_UF","UC_SEC_UF"))

design <- model.matrix( ~0+group)

design

y$samples$group <- group

y$samples

**#calculating counts per million (cpm) and normalizing voom transforming)**

mycpm <- cpm(list)

head(mycpm)

y$samples$lib.size

thresh <- mycpm > 0.5

table(rowSums(thresh))

keep <- rowSums(thresh) >= 2

summary(keep)

x <- y[keep, keep.lib.sizes=FALSE]

x$samples$lib.size

barplot(y$samples$lib.size/1e06, names=colnames(y), las=2, ann=FALSE, cex.names=0.75)

mtext(side = 1, text = "miRNA Samples", line = 4)

mtext(side = 2, text = "Library size (millions)", line = 3)

title("Barplot of library sizes")

logcounts <- cpm(x,log=TRUE)

boxplot(logcounts, xlab="", ylab="Log2 counts per million",las=2)

abline(h=median(logcounts),col="blue")

title("Boxplots of logCPMs (unnormalised)")

mtext(side = 1, text = "miRNA Samples", line = 4)

plotMDS(logcounts)

title("MDS plot for logcpm counts(unnormalized)")

z <- calcNormFactors(x)

z$samples

par(mfrow=c(1,2))

plotMD(logcounts,column = 1)

abline(h=0,col="red")

v <- voom(z,design,plot = TRUE)

names(v)

v

par(mfrow=c(1,2))

boxplot(logcounts, xlab="", ylab="Log2 counts per million",las=2,main="Unnormalised logCPM")

abline(h=median(logcounts),col="blue")

boxplot(v$E, xlab="", ylab="Log2 counts per million",las=2,main="Voom transformed logCPM")

abline(h=median(v$E),col="blue")

write.csv(v,"normalized - voom transformed logCPM for miRNA_methods.csv")

**# Make the DGEList**

**cur_DGELIST <- DGEList( counts=matrix_filter, group=group, lib.size=colSums( matrix_filter ) )**

**cur_DGELIST <- calcNormFactors( cur_DGELIST )**

**plotMDS( cur_DGELIST, method="bcv", col=as.numeric( cur_DGELIST$samples$group ) )**

**legend( "topright", as.character( unique( cur_DGELIST$samples$group )), col=c(1,4,2,3,5), pch=20)**

**#estimate dispersion for GLM**

**cur_DGELIST <- estimateGLMCommonDisp( cur_DGELIST, design )**

**cur_DGELIST <- estimateGLMTrendedDisp( cur_DGELIST, design )**

**plotMDS( cur_DGELIST, method="bcv", col=as.numeric( cur_DGELIST$samples$group ) )**

**legend( "topright", as.character( unique( cur_DGELIST$samples$group )), col=1:3, pch=20)**

**cur_DGELIST <- estimateGLMTagwiseDisp( cur_DGELIST, design )**

**plotMDS( cur_DGELIST, method="bcv", col=as.numeric( cur_DGELIST$samples$group ) )**

**legend( "topright", as.character( unique( cur_DGELIST$samples$group )), col=1:3, pch=20)**

**#filtering and making comparisons**

**fit <- glmFit( cur_DGELIST, design )**

**contrast_groups<- glmLRT(fit, contrast=makeContrasts( group**BPControl**-groupSEC_UC-groupSEC_UF-groupUC_SEC-groupUC_SEC_UF, levels=design ) )**

contrast_groups2<- glmLRT(fit, contrast=makeContrasts(groupBPControl-groupUC_SEC, levels=design ) )

contrast_groups3<- glmLRT(fit, contrast=makeContrasts(groupBPControl-groupSEC_UC, levels=design) )

contrast_groups4<- glmLRT(fit, contrast=makeContrasts(groupBPControl-groupSEC_UF, levels=design ) )

contrast_groups5<- glmLRT(fit, contrast=makeContrasts(groupBPControl-groupUC_SEC_UF, levels=design ) )

*#Followed same codes below for contrast_groups2-5 to get heatmaps. Changed highlighted parts accordingly.*

topTags( contrast_groups, n = 10 )

All_miRNA_comparison_contrast_groups <- topTags( contrast_groups, n = 2096 )

head(All_miRNA_comparison_contrast_groups)

write.csv(All_miRNA_comparison_ contrast_groups, "All_miRNA_comparison_contrast_groups_methods.csv")

**DGE analysis & create heatmap.**

dt_significant <- decideTestsDGE( contrast_groups, adjust.method="BH", p.value=0.05)

vctr_names_sig <- rownames( cur_DGELIST )[ as.logical( dt_significant )]

plotSmear( contrast_groups, de.tags = vctr_names_sig, cex=1 )

abline( h = c( -2, 2 ), col = "blue")

title ("Smear plot of contrast_groups ")

vctr_names_top <- rownames( topTags( contrast_groups, n=50))

vctr_sig <- as.logical( decideTestsDGE( contrast_groups, adjust.method="BH", p.value=0.05) )

vctr_names_hcl <- rownames( cur_DGELIST )[ vctr_sig ]

mtrx_significant <- cur_DGELIST$counts[ vctr_names_top, ]

vctr_colors = as.factor( c( "black", "red"))

vctr_sample_colors <- as.character( vctr_colors[ as.numeric( cur_DGELIST$samples$group ) ] )

mypalette <- brewer.pal(11,"RdYlBu")

morecols <- colorRampPalette(mypalette)

heatmap.2( log2( mtrx_significant), ColSideColors=vctr_sample_colors, key=TRUE, trace="none", col=rev(morecols(50)),main="Top 50 DE miRNA – sEV isolation methods ", scale="row", cexCol=1, cexRow=1.1, margins=c(7,8))

*******miRNA DE only in sEV compared to plasma (Figure 4a)*******

EXO <- read.csv("EXO only miRNA.csv", header=TRUE)

EXO_filter<- EXO[,-(1)]

rownames(EXO_filter) <- EXO[,1]

list2 <- EXO_filter

z <- DGEList(list2)

vctr_names_top <- rownames(z)

vctr_names_hcl <- rownames( z )[ vctr_names_top ]

mtrx_significant3 <- z$counts[ vctr_names_top, ]

vctr_colors = as.factor( c( "black", "red","blue","green","yellow"))

vctr_sample_colors <- as.character( vctr_colors[ as.numeric( cur_DGELIST$samples$group ) ] )

heatmap.2( log2( mtrx_significant3 _ 1 ), ColSideColors=vctr_sample_colors, key=TRUE, trace="none", col=rev(morecols(50)),main="miRNA DE in sEV

contrast to blood plasma",, scale="row", cexCol=1, cexRow=1.1, margins=c(7,8))

***********Plasma only top 25 miRNA (Figure 5a) **********

Plasma <- read.csv("Plasma only top 25 miRNA.csv", header=TRUE)

Plasma_filter<- Plasma[,-(1)]

rownames(Plasma_filter) <- Plasma[,1]

list3 <- Plasma_filter

r <- DGEList(list3)

vctr_names_top <- rownames(r)

vctr_names_hcl <- rownames( r)[ vctr_names_top ]

mtrx_significant4 <- r$counts[ vctr_names_top, ]

vctr_colors = as.factor( c( "black", "red","blue","green","yellow"))

vctr_sample_colors <- as.character( vctr_colors[ as.numeric( cur_DGELIST$samples$group ) ] )

heatmap.2( log2( mtrx_significant4 _ 1 ), ColSideColors=vctr_sample_colors, key=TRUE, trace="none", col=rev(morecols(50)),main="miRNA DE in Plasma

(top 25

contrast to sEV)",, scale="row", cexCol=1, cexRow=1.1, margins=c(7,9))
